# Supercharging CAR-T cells through transcriptional and epigenetic armoring

**DOI:** 10.7150/thno.107908

**Published:** 2025-02-18

**Authors:** Diyuan Qin, Yanna Lei, Pei Shu, Yugu Zhang, Yuin-Han Loh, Yongsheng Wang, Qijing Li

**Affiliations:** 1 Cancer Center, Clinical Trial Center, West China Hospital, Sichuan University, Chengdu, Sichuan, China.; 2 Institute of Molecular and Cell Biology (IMCB), Agency for Science, Technology and Research (A ∗ STAR), Singapore 138673, Singapore.; 3 Division of Thoracic Tumor Multimodality Treatment, Cancer Center, West China Hospital, Sichuan University, Chengdu, Sichuan, China.; 4 Division of Abdominal Tumor Multimodality Treatment, Cancer Center, West China Hospital, Sichuan University, Chengdu, Sichuan, China.; 5 Cancer Center, National Medical Products Administration Key Laboratory for Clinical Research and Evaluation of Innovative Drugs, West China Hospital, Sichuan University, Chengdu, Sichuan, China.; 6 Singapore Immunology Network (SIgN), Agency for Science, Technology and Research (A*STAR), Singapore 138648, Singapore.; 7 Department of Microbiology and Immunology, Yong Loo Lin School of Medicine, National University of Singapore, Singapore 117545, Singapore.

**Keywords:** CAR-T cells, transcriptional regulation in immunotherapy, epigenetic modification, exhaustion and memory differentiation, tumor microenvironment

## Abstract

Inspired by the remarkable success of CAR-T therapy in hematologic malignancies, research is increasingly focused on adapting this treatment for solid tumors. However, CAR-T efficacy remains limited due to its exhaustion and shortened persistence. Transcription factors and epigenetic modifications play pivotal roles in modulating T cell differentiation and functionality, which have been leveraged in numerous strategies to promote the formation of long-lasting memory cells with stem-like properties and supercharging CAR-T performance. This review highlights pivotal transcriptional factors, such as c-Jun and FOXO1, which enhance and sustain T cell effector function, diminishes exhaustion, and epigenetic regulators like TET2 and DNMT3A, whose knockout promotes memory T subsets formation. We explore their interconnections, downstream targets, biological impacts, and the potential application risks of certain candidates, providing a comprehensive theoretical framework for supercharging CAR-T therapies through transcriptional and epigenetic interventions.

## 1. Introduction

Chimeric antigen receptor (CAR) T cells are genetically engineered to express a receptor on cell membrane that consists of an antibody fragment capable of specifically recognizing tumor antigens, coupled with intracellular costimulatory and activation domains that trigger T cell activation and function. This innovation facilitates precise tumor antigen targeting, robust costimulatory signaling, and the production of effector molecules, such as interferon-γ, granzyme B, and perforin, that effectively eradicate tumor cells. CAR-T therapy has achieved significant clinical success in treating hematologic malignancies 1-4. However, their therapeutic efficacy in solid tumors remains suboptimal due to various inhibitory factors 5, 6.

The immunosuppressive cells and cytokines in the tumor microenvironment 7, along with abnormal tumor vasculature and metabolic competition, directly suppress CAR-T cell activity 8-11. Additionally, chronic exposure to tumor antigens frequently induces T cell exhaustion, characterized by diminished proliferation and attenuated effector cytokine production, which compromises durable memory T cells formation, undermines sustained antitumor responses and ultimately results in tumor relapse and treatment failure 12.

T cell differentiation status directly influences the antitumor efficacy of immunotherapy 13-15. Memory T cells are key players in maintaining long-term protective immunity, enabling the immune system to launch faster and more robust reactions to previously encountered antigens 16, 17. The persistence of durable memory T cells is closely associated with improved CAR-T efficacy and extended disease remission 18-21. Numerous studies are seeking strategies to prolong T cell persistence while alleviating exhaustion, both of which are crucial for enhancing antitumor responses 12, 22.

Recent advances in research have revealed that T cell memory differentiation is governed by the interaction of transcription factors (TFs) and epigenetic modifications. TFs orchestrate gene regulatory networks and serve as convergence points for signaling pathways driving T-cell differentiation 23, 24. Epigenetic modifications such as DNA methylation, histone acetylation, and chromatin remodeling regulate gene expression and are also essential for T cell differentiation.

Importantly, TFs and epigenetic regulation are tightly interdependent: TFs recruit epigenetic complexes to modulate chromatin state, while epigenetic changes alter chromatin accessibility and impact TFs activity 25-27. Together, they coregulate gene expression, forming a core mechanism that governs T cell commitment in response to environmental signals. Targeting TFs and epigenetic regulators offers a promising strategy to boost CAR-T cell expansion, stemness and resistance to exhaustion, ultimately strengthening CAR-T efficacy 12.

This review aims to summarize and highlight the roles of crucial TFs and epigenetic regulators in CAR-T cells, as well as their connection and impact on therapeutic outcomes, underscoring the potential of targeting TFs and epigenetic mechanisms to advance adoptive cell immunotherapy.

## 2. TFs and epigenetic regulators cooperate in T cell differentiation

Elucidating the mechanisms governing T cell fate decisions is significant for optimizing immunotherapies by precisely modulating T cell states. Advances in next-generation sequencing (NGS) technologies, such as ATAC-seq and scRNA-seq, enable comprehensive and detailed analysis of transcriptional changes across various T cell subsets. Numerous critical TFs and epigenetic regulators contributing to T cell memory differentiation have been identified.

TFs cooperate with epigenetic regulators to coordinate T cell differentiation. They directly control downstream gene transcription while shaping the epigenetic landscape through their intrinsic epigenetic regulatory functions or by recruiting chromatin-modifying enzymes, such as histone acetyltransferases or methyltransferases. Interferon regulatory factor 4 (IRF4) is crucial for driving naïve CD8^+^ T cells activation, expansion and effector differentiation. Together with basic leucine zipper ATF-like transcription factor (BATF) and Jun family members, IRF4 drives key TFs expression such as Runt-related transcription factor 3 (Runx3), T-box expressed in T cells (T-bet) and B-lymphocyte-induced maturation protein 1 (Blimp-1) while upregulating the expression of effector cytokines and receptors, which are essential for T cell effector functions and differentiation 28, 29. Prior to the first division of naïve T cells, Runx3 initiates chromatin accessibility changes that open memory T cell-specific cis-regulatory regions. These regions are highly enriched with binding motifs for *bZIP*, *IRF* and *Prdm1*, leading to IRF4 and Blimp-1 upregulation and facilitating early effector and memory precursor cell differentiation 30. Blimp-1 further recruits histone-modifying enzymes, including histone deacetylase 2 (HDAC2) and G9a, to target loci such as *Il2ra* and *Cd27*, causing increased histone H3K9 trimethylation and decreased histone H3 acetylation, which leads to the downregulation of target gene transcription 31. Other TFs, such as lymphoid enhancer-binding factor 1 (LEF1) and T cell factor 1 (TCF1), display HDAC activity during T cell development, inhibiting histone acetylation in T cells to establish an epigenetic signature conducive to CD8^+^ T cell identity 32.

Moreover, epigenetic modifications also affect TFs expression. The upregulation of *de novo* methyltransferase DNA (cytosine-5)-methyltransferase 3A (DNMT3A) in activated T cells leads to *Ccr7*, *Tcf7*, and *Sell* methylation, which results in decreased gene transcription 33, 34.

T cell activation triggers dynamic variations in chromatin accessibility across differentiation stages, resulting in unique chromatin accessibility profiles. These changes are closely tied to the binding of specific TFs, reflecting the identical function and development states of T cells. For instance, TCF1 binding sites are prevalent in memory and naïve T cells, basic leucine zipper domain protein (bZIP), T-box and IRF motifs are enriched in effector and memory subsets, while chromatin regions enriched with nuclear receptor subfamily 4A (NR4A) and nuclear factor of activated T cells (NFAT) motifs are hallmarks of exhausted T cells 35.

Yu *et al.* explored TFs crucial for effector or memory CD8^+^ T cell commitment in *Listeria* infected mice. Effector T cell subsets are enriched with motifs binding T-bet, BATF, sterol regulatory element binding protein 2 (SREBP2) and AP-1 36, which are pivotal for CD8^+^ T cells activation and functional capacity 29, 37-39. Conversely, motifs associated with TCF1, LEF1, and E2A are predominantly enriched in naïve, memory precursor, and memory T cell subsets, correlating with their roles in maintaining stem-like properties and long-term immune memory 40-42.

Therefore, decoding the regulatory networks between TFs and epigenetic regulators will aid in understanding T cell differentiation. This knowledge provides a foundation for fine-tuning T cell differentiation, thereby optimizing T cell-based immunotherapies.

## 3. Therapeutic targeting of transcription factors in CAR-T therapy

T cell differentiation and functionality are orchestrated by complex gene regulatory networks, with TFs playing a pivotal role in this process. In our review, we categorized key TFs into two groups based on their impact: those promoting memory differentiation and those inducing exhaustion. Memory-promoting TFs, such as c-Jun 43, FOXO1 44, 45 and FOXP1 46, play critical roles in maintaining T cell stemness and resilience against exhaustion. The overexpression of these TFs has been shown to enhance the proliferative capacity, improve antitumor activity, and limit terminal differentiation in CAR-T cells. In contrast, TFs such as BATF 47, thymocyte selection-associated high mobility group box (TOX) 48, Blimp-1 49 and the NR4A family 50 are linked to T cell exhaustion and dysfunction. Knockout of these TFs has been demonstrated to downregulate exhaustion-associated genes, promote memory T cell formation, and strengthen antitumor efficacy (**Table 1**).

### 3.1 Transcription factors positively correlated with functional CAR-T cells

Numerous TFs have been identified that support memory phenotype maintenance and boost CAR-T cell effector functionality. Overexpressing these TFs in CAR-T cells has demonstrated potential in counteracting exhaustion and upgrading immunotherapies.

#### 3.1.1 c-Jun

**Figure 1:**
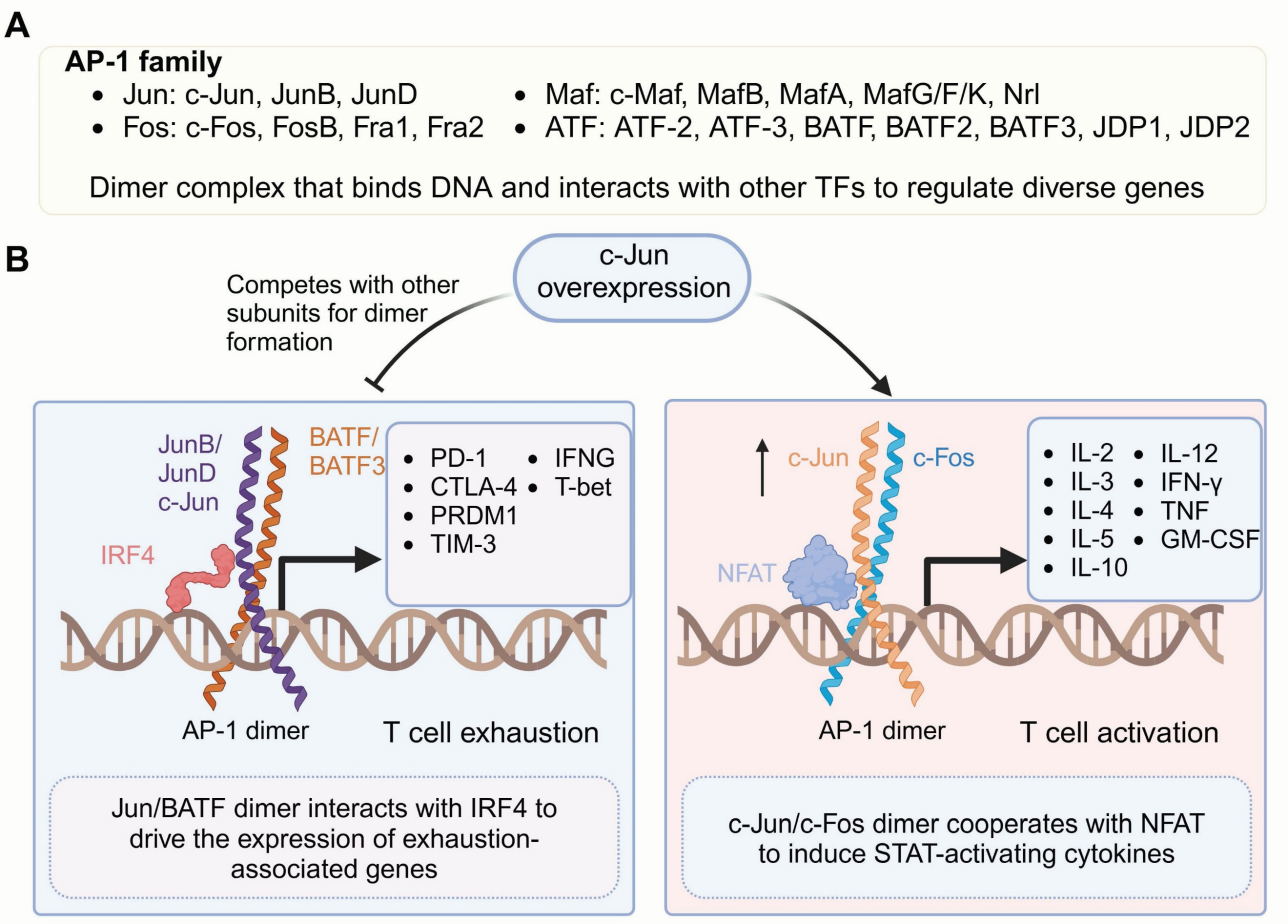
** Family members of AP-1 and their roles in regulating gene transcription.** (A) Members of the AP-1 TF family. Active AP-1 is a dimeric transcription factor complex (Jun, Fos, ATF, BATF, JDP2) that binds DNA with partner-specific preferences and interacts with other TFs to form ternary complexes targeting composite DNA motifs. (B) Overexpressed c-Jun, together with c-Fos, promotes high-affinity AP-1 dimer formation, reducing the formation of dimers involving other subunits. The c-Jun/c-Fos dimer complexes with NFAT drive the expression of genes critical for T cell activation, including cytokines associated with STAT signaling, such as IL-2, IL-4, and IL-12. In contrast, under exhaustion-inducing conditions, the BATF/JUN heterodimer forms a complex with IRF4. This complex binds to specific DNA motifs, promoting the transcription of genes associated with T cell exhaustion, thereby contributing to reduced T cell functionality. Figure was created in BioRender.com.

c-Jun is a member of the nuclear transcription activator protein-1 (AP-1) family (**Figure 1**), which possesses a conserved basic DNA-binding domain and a bZIP region. The bZIP mediates subunits from the Jun, Fos, Maf or activating transcription factor (ATF) families form AP-1 homodimers or heterodimers, enabling specific binding to target DNA sequences by relying on the basic domain. The composition and complex abundance of the AP-1 dimer dictate its specificity and function in gene activation or repression and regulating critical processes, including activation, proliferation, transformation, migration, inflammation and apoptosis 82-85.

Jun and Fos proteins represent the core components of AP-1 and are its most extensively studied subunits. The heterodimer complex formed by Jun/Fos has a stronger DNA-binding affinity compare to the Jun/Jun homodimer, thereby resulting in greater transcriptional activation potential 86, which drives the transcription of IL-2, a crucial cytokine essential for T-cell activation and expansion 87. Moreover, AP-1 dimers can associate with other TFs to form ternary complexes, enabling them to bind specific DNA motifs and modulate gene expression. Specifically, the Jun/Fos heterodimer has been shown to form a ternary complex with NFAT, which drives CD8^+^ T cell activation and serials of cytokines secretion, such as IL-3, IL-4, IL-5, IL-10, IL-12, interferon γ (IFN-γ), tumor necrosis factor (TNF) and granulocyte-macrophage colony-stimulating factor (GM-CSF) 88-90, which cooperate to sustain T cell effector functions. However, with prolonged antigen stimulation and the absence of costimulatory signals, AP-1 is progressively downregulated, leaving NFAT alone to drive T cell exhaustion and promote the upregulation of inhibitory receptors, including cytotoxic T-lymphocyte-associated protein 4 (CTLA-4), LAG3, programmed cell death protein 1 (PD-1) and TIM-3 91-94.

In 2019, Mackall and colleagues discovered that c-Jun overexpression strengthens CAR-T cells efficacy and counteracts dysfunction 43, 95. HA-28z CAR-T cells served as a model to explore T cell exhaustion, revealing the pivotal role of AP-1-associated bZIP/IRF families in this process. c-Jun outperforms other AP-1 subunits, forming c-Jun/c-Fos dimers and reducing JunB/BATF dimerization as well as downregulating JunB, BATF, and BATF3 protein levels. c-Jun overexpression prevents T cells excessively expressing immunoregulatory receptors and terminal differentiation, resulting in an increased memory population and enhanced antitumor activity. In a leukemia mouse model, Feng and colleagues showed that c-Jun triggers the extracellular signal-regulated kinase1/2 (ERK1/2) cascade, boosts the co-stimulatory signaling 51, increases IL-2 and IFN-γ secretion, and positively regulates cell activation and T cell effector functions without upregulating exhaustion-related genes. A phase I clinical trial in which c-Jun overexpressing CD33 CAR-T cells were used to treat acute myeloid leukemia confirmed that c-Jun significantly boost CAR-T effector activity. Among all four patients who successfully underwent CAR-T therapy, substantial CAR-T cells expansion and clinical benefit were observed 51. These results highlight the significant potential of c-Jun in augmenting CAR-T cell therapeutic efficacy.

However, c-Jun overexpression significantly activates CAR-T cells, which may trigger massive cytokines release and pose a risk of severe therapeutic side effects. In the aforementioned clinical trial, the study was prematurely halted due to severe adverse events, including grade 4 cytokine release syndrome (CRS) and infection, observed in the second patient 51. Moreover, a clinical trial of receptor tyrosine kinase-like orphan receptor 1 (ROR1) CAR-T cells overexpressing c-Jun revealed a potential risk of treatment-related pneumonia in cancer patients with lung metastases. One participant experienced Grade 5 respiratory failure 96. Therefore, while the overexpression of c-Jun can enhance CAR-T effector activity, it is crucial and necessary to be aware of the associated potential toxic side effects.

#### 3.1.2 TCF1

*TCF7* is located on chromosome 5 and encodes the human transcription factor TCF1. Among the reported isoforms, full-length TCF1 contains an N-terminal domain that interacts with β-catenin 52, which is involved in Wnt signaling regulation. This process is followed by a histone deacetylase (HDAC) activity segment, which is essential for defining CD8^+^ T cell lineage and preserving a less differentiation state. The C-terminal region includes a high-mobility-group (HMG) box DNA-binding domain, which governs gene transcription by binding to enhancers and promoters 32. In contrast, the short isoform lacks the β-catenin-binding domain but retains the HMG and HDAC activity domains 97. Interestingly, the TCF1 homolog LEF1 also has similar DNA-binding domains and HDAC activity sequences, positioning it as a critical TF closely associated with T cell development and differentiation 40, 52, 98.

**Figure 2:**
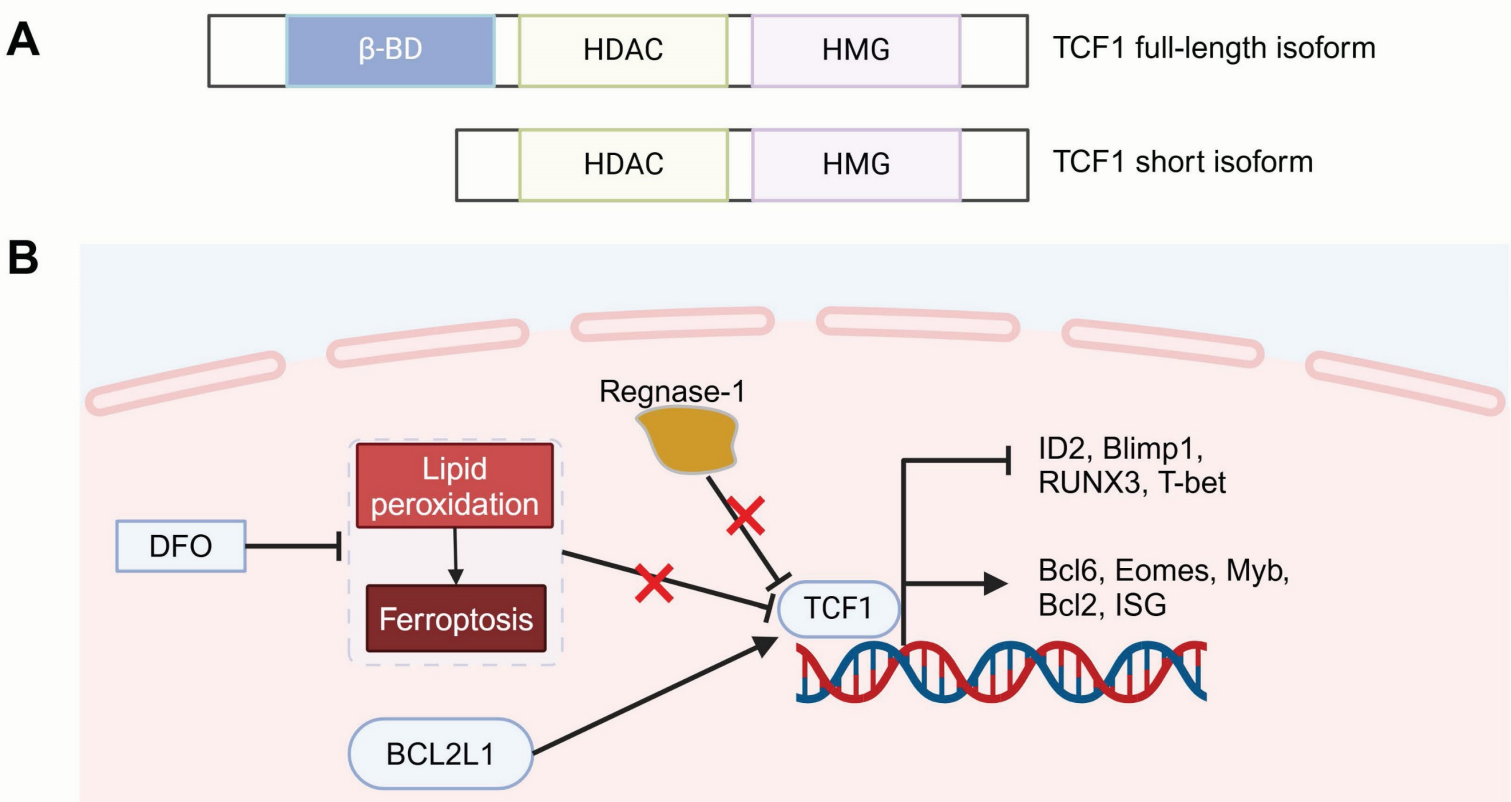
** Structure of TCF1 and strategies for regulating T cell memory differentiation.** (A) TCF1 has two isoforms: a full-length isoform (containing the β-catenin interacting domain, HDAC activity segment, and HMG DNA-binding domain) and a short isoform lacking the β-catenin binding domain. (B) Strategies such as regnase-1 deficiency, DFO treatment to mitigate lipid peroxidation and ferroptosis, and BCL2L1 overexpression for optimizing CAR-T therapy by modulating TCF1 expression to promote T cell memory differentiation. HDAC: histone deacetylase; HMG: high-mobility-group box; DFO: deferoxamine. Figure was created in BioRender.com.Transcriptomic analysis showed that CAR-T cells from complete responders with chronic lymphocytic leukemia (CLL) were enriched with memory-promoting genes, such as *TCF7* and *LEF1* 18. Moreover, Zheng and colleagues suggest that upregulating TCF1 expression could help to form a desirable immunological output 101. Regnase-1 was identified as a critical regulator of CAR-T cell differentiation, directly suppressing *Tcf7* and hindering the development of immune-supporting precursor exhausted T (Tpex) cells. Regnase-1 deficiency could promote TCF1 expression, thereby enhancing CAR-T cells persistence and achieving sustained tumor control. Teshima and colleagues used the iron chelator deferoxamine (DFO) during the production of mouse CAR-T cells to reduce lipid peroxidation and inhibit ferroptosis 102. This not only increased the proportion of TCF1^+^TIM-3^-^ progenitor subsets, which are known to be linked to the longevity of CAR-T but also enhanced CAR-T cell cytotoxicity and *in vivo* antitumor effects. Another study involved overexpressing the BCL2 Like 1* (BCL2L1)* gene in B-cell maturation antigen (BCMA) targeting CAR-T cells 103. These BCMA_BCL2L1 CAR-T cells exhibited higher TCF1 and lower TOX/TOX2 expression, showed an increased presence of naïve T cells and less-differentiated T cells, such as early activated T cells and precursor T cells, which improved the functional versatility of CAR-T cells and boosted their proliferation and cytotoxicity under chronic antigen stimulation *in vitro*, achieving excellent tumor clearance in a multiple myeloma (MM) xenograft model.

Recent advances have highlighted TCF1 as a versatile regulator, exerting context-dependent functions that shape CAR-T cell phenotypes across varying conditions and stages of T cell differentiation. Upon chronic antigen exposure, CD8^+^ T cells differentiate into TCF1^high^ progenitor-like subsets and TCF1^low^ terminal subsets 52. TCF1 suppresses several pro-exhaustion factors, such as Blimp-1, TIM-3, Tbx21, ID2 and Runx3, while maintaining T cell stemness by upregulating memory-related genes, like EOMES, cMyb and interferon-stimulated gene (ISG), while reinforcing the TCF1-Bcl6 axis 99. TCF1 is essential for the memory-subset formation and serves as a predictor of the responses to immune checkpoint blockade (ICB) and tumor-infiltrating lymphocyte (TIL) therapy 52, 100. Considering its significant role in T cell immunity, leveraging TCF1 and its downstream transcriptional pathways holds potential to strengthen the antitumor effectiveness of CAR-T cells (**Figure 2**).

However, the strategy of enhancing the CAR-T memory phenotype by directly overexpressing TCF1 remains debatable. Weber and colleagues directly overexpressed the *TCF7* gene, which indeed increased oxidative phosphorylation (OXPHOS) and metabolic adaptability in CAR-T cells but failed to promote the expansion of memory subsets 45. Transcriptome sequencing revealed that TCF1 overexpressing cells exhibited high levels of TOX, CD39 and NR4A family exhaustion-related TFs. Further mechanistic studies are needed to uncover the regulatory networks involving TCF1 and its role in T cell memory formation and subsequently adapt it to optimize CAR-T antitumor efficacy.

#### 3.1.3 FOXO1

Forkhead box protein O1 (FOXO1), belonging to the forkhead TF family, participants in regulating cell metabolism, the regulation of cell division, programmed cell death, genomic repair processes, and the response to oxidative stress 104. Structurally, FOXO1 contains a conserved DNA-binding domain (DBD) that facilitates its interaction with specific target genes, a nuclear localization signal (NLS) responsible for its nuclear import, a nuclear export signal (NES) that regulates its cytoplasmic translocation, and a transactivation domain (TAD) that mediates its transcriptional activity. Two independent papers published in 2024 highlighted FOXO1 as a vital regulator of CAR-T cell stemness, efficacy as well as metabolic fitness 44, 45. FOXO1 activity is primarily controlled through protein modifications, including phosphorylation, acetylation, ubiquitylation and methylation 105. The PI3K-AKT signaling pathway serves as a primary regulator of FOXO1 activity by promoting its phosphorylation through AKT. This phosphorylation exposes the NES on FOXO1, facilitating its translocation from the nucleus to the cytoplasm, thereby hindering its transcriptional activity 106. The functions of FOXO1 within immune cells are also progressively being elucidated. FOXO1 regulates the homeostasis and survival of naïve T cells by controlling interleukin 7 receptor (IL-7R), L-selectin (CD62L) and C-C chemokine receptor type 7 (CCR7) 107, 108. Moreover, FOXO1 also regulates the activity and functions of dendritic cells 109, macrophages 110 and B cells 111.

**Figure 3:**
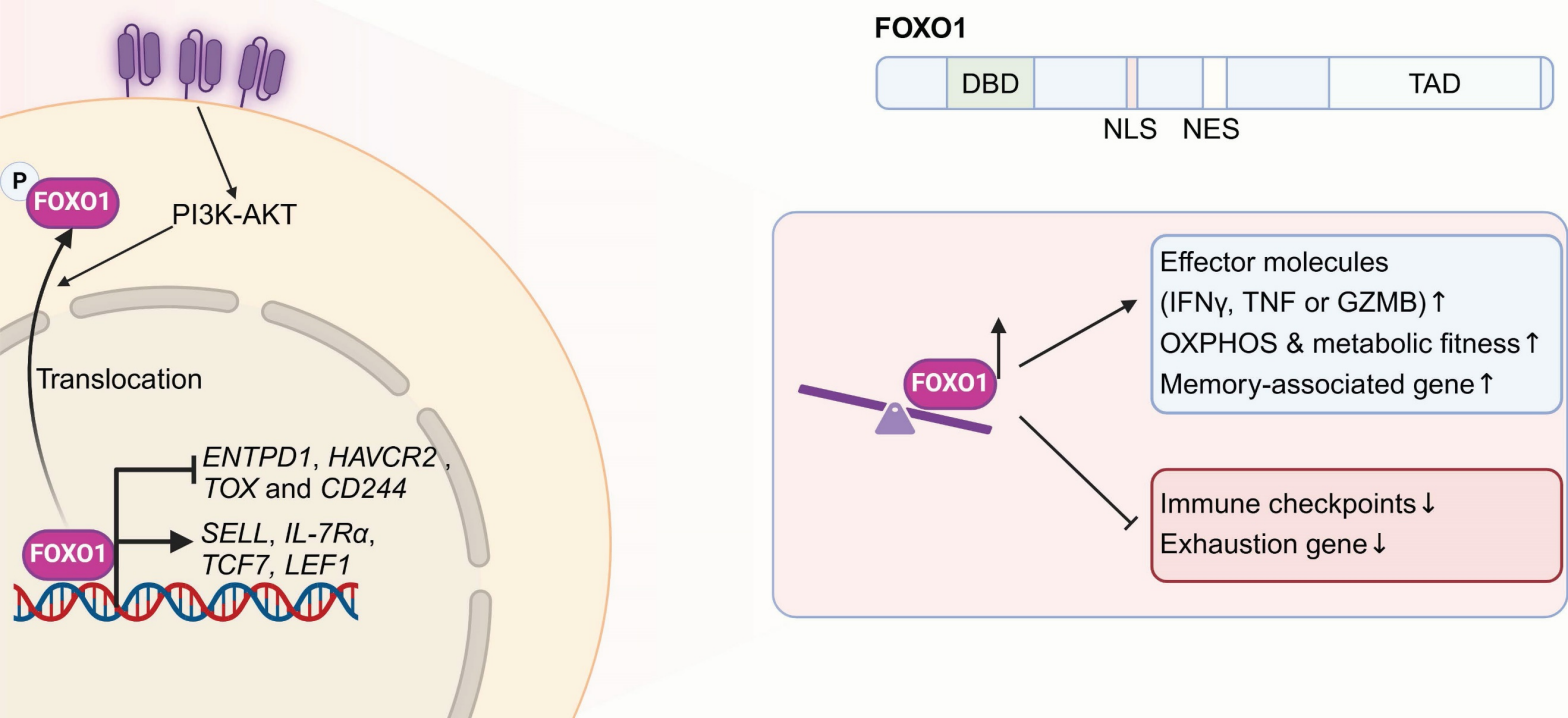
** Overexpressing FOXO1 promotes CAR T cell memory formation, improves metabolic adaptability, and boosts antitumor effectiveness.** The FOXO1 structure includes a conserved DNA-binding domain (DBD), nuclear localization (NLS) and export signals (NES) for nuclear-cytoplasmic transport, and a transactivation domain (TAD) for transcriptional activity. The PI3K-AKT pathway regulates FOXO1 by promoting its AKT-mediated phosphorylation, exposing its NES and driving nuclear export, which inhibits transcriptional activity. FOXO1 overexpression in CAR-T cells promotes a stem-like phenotype, metabolic fitness, and enhanced cytokine secretion by upregulating memory-associated genes (SELL, IL-7Rα, TCF7 and LEF1) and suppressing exhaustion-related genes (CD39, HAVCR2, TOX, CD244). Figure was created in BioRender.com.

Here, both research teams aimed to boost CAR-T cell expansion and persistence *in vivo*, pinpointing FOXO1 as a critical target for resisting T cell exhaustion and enhancing therapeutic efficacy (**Figure 3**) 44, 45. Compared with TCF7 or ID3, FOXO1 overexpression proved to be the most effective in maintaining the stemness of human Lewis Y CAR-T cells. It significantly reduced chromatin accessibility at regions rich in AP-1 and NF-κB specific motifs, thereby promoting a quiescent T cell state by inhibiting AP-1 activity. FOXO1 overexpression in CAR-T cells promoted a CD45RA^+^CD62L^+^ stem-like phenotype, metabolic fitness, and enhanced cytokine secretion by promoting the expression of memory-differential genes (SELL, IL-7Rα, TCF7, and LEF1) and suppression of exhaustion-related genes (CD39, HAVCR2, TOX, and CD244). In both leukemia and solid tumors, FOXO1 overexpression prolonged CAR-T cell persistence and strengthened tumor control capabilities. Furthermore, transcriptomic analyses of CAR-T clinical data revealed marked enrichment of FOXO1 regulon downstream genes in pre-infusion CAR-T products from CLL patients with complete or partial responses, correlating with *in vivo* CAR-T expansion and overall survival. Epigenetic profiling further demonstrated that T cells from patients with durable CAR-T responses were enriched with FOXO1-associated epigenetic signatures. Thus, FOXO1 overexpression presents a promising approach for improving the effectiveness of T cell-based cancer immunotherapies.

#### 3.1.4 STAT5

**Figure 4:**
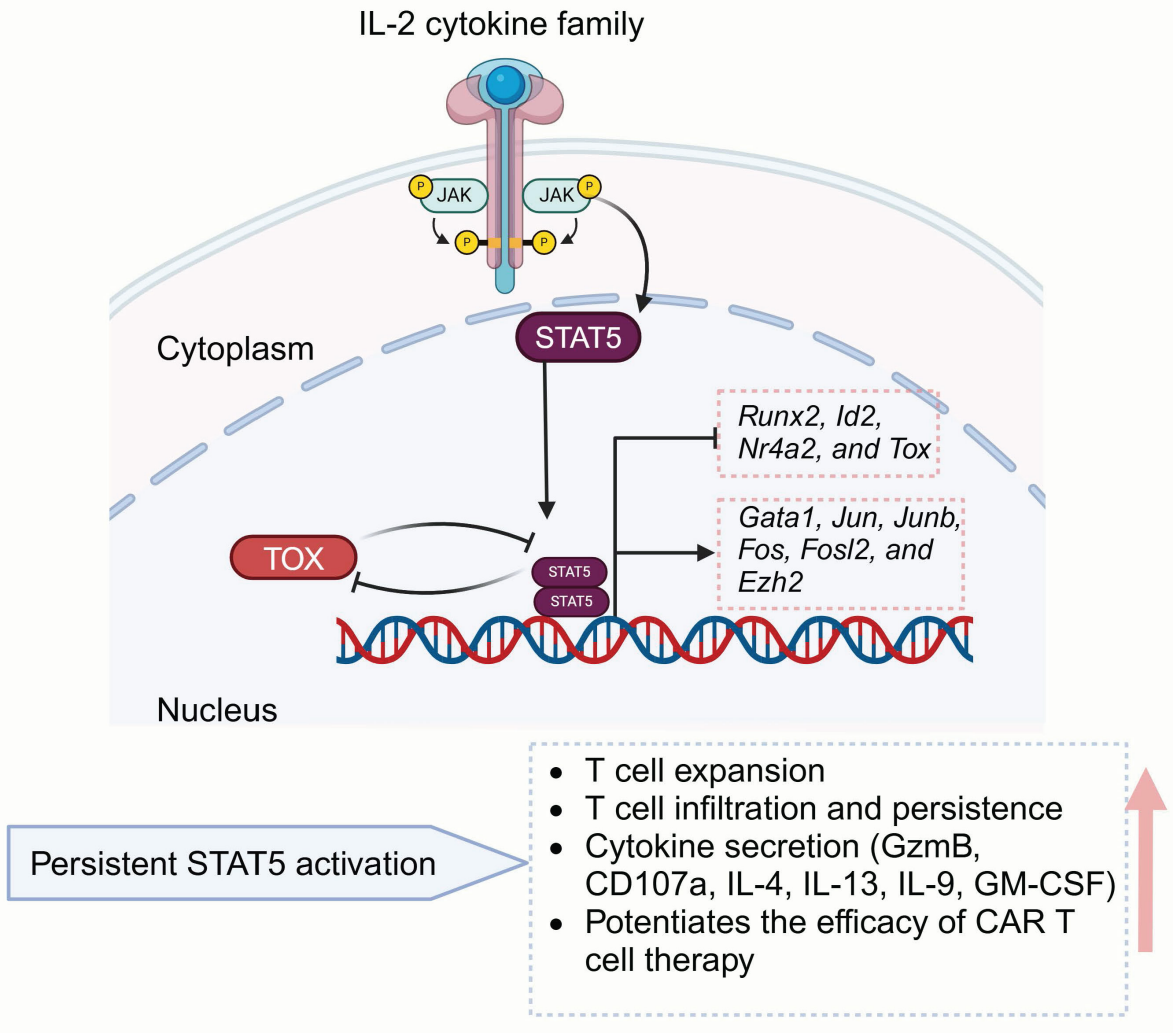
** Schematic of STAT5-mediated T cell regulatory mechanisms.** The IL-2 cytokine family promotes STAT5 expression, while STAT5 counteracts TOX to regulate T cell proliferation, cytokine secretion, and memory formation, ultimately enhancing CAR-T antitumor efficacy. Figure was created in BioRender.com.

Signal transducers and activators of transcription (STAT) typically refer to two closely related isoforms, STAT5a and STAT5b, which exhibit 94% structural similarity 112. STAT5 takes part in numerous vital cellular processes^113^, ^114^, especially in regulating T cell homeostasis as well as immune function (**Figure 4**) 115, 116. The activity of STAT5 proteins is predominantly regulated by cytokines from the IL-2 family, such as IL-2, IL-4, IL-7, IL-9, and IL-15. These cytokines utilize a shared gamma chain (γ(c)) within their receptor complexes 117-119 to induce downstream signals.

STAT5 functionally rejuvenates exhausted CD8^+^ T cells by counteracting TOX and reshaping the terminal differentiation epigenic landscape, thereby enhancing the response to ICB treatment 53, 54. CD4^+^ T cells also closely influence T cell effectiveness via inflammatory cytokines releasing and orchestrating multiple immunologic effector mechanisms 120, 121. IL-7, a gamma-chain cytokine, is widely utilized in *ex vivo* T cell cultures in CAR-T manufacturing due to the ability to enhance the stemness and polyfunctionality of CD4^+^ T cells through STAT5 activation 122, 123. Directly modulating STAT5 could also enhance CAR-T cells polyfunctionality. Ding *et al.* engineered CD4^+^ T cells to express a variant of STAT5 with constitutive activity (CASTAT5) 55, demonstrating that these modified cells possess enhanced expansion potential following adoptive transfer and secrete multiple helper T cell (Th) cytokines, including Granzyme B (GzmB), CD107a, IL-4, IL-9, IL-13 and GM-CSF. Moreover, CASTAT5 significantly promotes CD4^+^ T cell tumor infiltration, which supports CAR-T cell proliferation and persistence, while enhancing overall effector function in a CD8^+^ T cell-dependent manner. Next-generation sequencing revealed that CASTAT5-producing CD4^+^ T cells undergo extensive remodeling of their transcriptional and epigenetic landscapes. STAT5A activates genes such as *Junb*, *Jun*, *Fos*, *Fosl2*, *Ezh2* and* Gata1*, while inhibiting *Id2*, *Nr4a2*, *Runx2*, and *Tox*. CASTAT5 expression also benefits CD8^+^ T cells, and its co-expression in both CD4^+^ and CD8^+^ T cells results in the most pronounced antitumor effects. Thus, sustained STAT5 activation in CAR-T cells presents a promising strategy to improve therapeutic effectiveness.

#### 3.1.5 FOXP1 and KLF2

**Figure 5:**
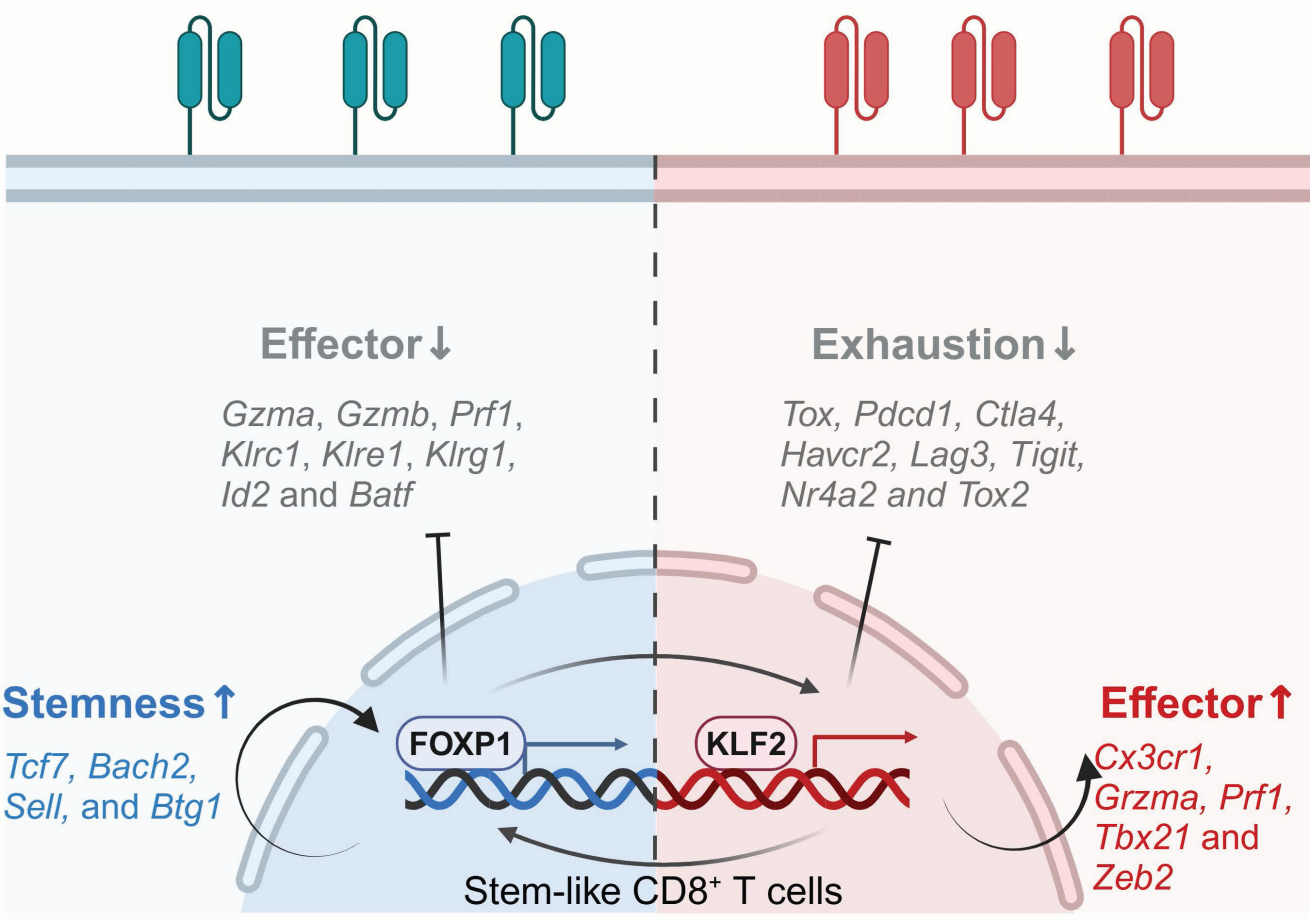
** FOXP1 and KLF2 are master transcription factors that govern CAR-T cell fate and therapeutic efficacy.** FOXP1 preserves the quiescence and homeostasis of naïve T cells, limiting their transition into effector cells while supporting CAR-T cell stemness and enhancing antitumor activity. KLF2 acts as a pivotal TF within effector CD8^+^ CAR-T cell networks, promoting effector differentiation and enhancing resistance to exhaustion through suppressing exhaustion-related TFs and genes. Figure was created in BioRender.com.

Epigenomic and transcriptomic analyses have identified forkhead box P1 (FOXP1) and Kruppel-like factor 2 (KLF2) as key regulators of CD8+ CAR-T cell stemness and effector functionality, respectively 46. Zhu and colleagues uncovered the heterogeneous chromatin states of CAR T cells among solid tumors and leukemia. They clarified gene networks governing CAR-T differentiation, exhaustion and dysfunction, identifying master TFs that govern CAR-T fate and therapeutic outcomes. These two TFs exhibit reciprocal regulation, where FOXP1 deficiency reduces KLF2 expression, and vice versa (**Figure 5**) 124.

FOXP1, part of the Foxp subgroup within the forkhead TF family, is pivotal in immune responses, organ development and cancer pathogenesis 125-127, while also maintaining the quiescence and homeostasis of naïve T cells, partly by antagonizing FOXO1 interaction with shared forkhead-binding sites within the IL-7Rα enhancer, thus repressing IL-7Rα expression and MEK-Erk signaling. Consequently, FOXP1 deficiency leads to increased IL-7R expression in T cells, enabling enhanced proliferation upon IL-7 stimulation 126. Zhu and colleagues showed that FOXP1 deletion in CD8^+^ CAR-T cells downregulates stemness-associated genes such as *Tcf7*, *Bach2*, *Sell*, and *Btg1*, while upregulating effector-related genes including *Gzma*, *Gzmb*, *Prf1*, *Klrc1*, *Klre1*, *Klrg1, Id2 and Batf*. CD8^+^ CAR-T lacking FOXP1 impair tumor suppression and diminish the proliferative capacity and lower the expression of the memory-related markers 46. These findings validate FOXP1's role in supporting CAR-T cell stemness and enhancing anti-tumor activity.

KLF2, a member of the Krüppel-like TF family, regulates the differentiation, migration, functionality and homeostasis of immune cells, such as T and B lymphocytes, NK cells and monocytes 128-131, and participates in various biological processes in cancers 132, 133. KLF2 governs T cell trafficking by upregulating the lipid-binding receptor sphingosine-1-phosphate (S1P) and the selectin CD62L. In the absence of KLF2, impaired peripheral migration causes CD4/CD8 single-positive T cells to be retained within the thymus, resulting in a significant reduction of T cells in the peripheral blood, lymph nodes and spleen 134, 135. Besides regulating T cell migration, KLF2 facilitates the establishment of effector phenotypes in T cells. Knockout of KLF2 reduces the abundance of T effector-like cells, downregulates genes associated with effector characteristics, such as *Gzma, Klrc1, Klrd1, Zeb2, Prf1, Tbx21,* and* Cx3cr1*. Furthermore, it also upregulates inhibitory receptors expression, including *Pdcd1, Ctla4, Tigit, Havcr2* and* Lag3*, while promoting exhaustion-related TFs like *TOX2* and *NR4A2.* Additionally, it amplifies TOX-induced transcriptional signatures and downregulates TOX-repressed genes. Chromatin accessibility analysis revealed that T cells lacking KLF2 exhibited enhanced accessibility at the *Pdcd1* and *Tox* gene loci, as well as at the NFAT and AP-1 TF binding sites. These data suggest that KLF2 promotes T cell differentiation into effector subsets and inhibits exhaustion by regulating T cell transcription and epigenetics 46.

### 3.2 Transcription factors associated with CAR-T cells exhaustion

T cells exhaustion poses a significant hurdle for CAR-T therapy in solid tumors. Extensive research has focused on uncovering critical regulators responsible for T cell dysfunction 136, 137. Several exhaustion-inducing TFs have emerged as key targets, such as BATF, TOX, NR4A and Blimp-1. Recent studies suggest that knocking out these regulators holds significant potential for prolonging CAR-T cell functionality.

#### 3.2.1 BATF

BATF belongs to the AP-1 TF family and negatively regulates AP-1-mediated T cell effector functions by competing with Fos subunits for Jun binding, which leads to transcriptionally repressive Jun/BATF heterodimer formation 138, 139 (**Figure 1**). The Jun/BATF dimer further binds to IRF4 or IRF8 and specifically recognizes AP-1-IRF composite elements (AICEs) within target genes, facilitating CD8^+^ T cell exhaustion 138, 140. BATF also plays a positive transcriptional regulatory role in influencing T cell commitment and supporting the functional maturation of dendritic cells and B cells 141-145. However, the role of BATF in T cell function remains controversial because of conflicting findings from different research groups. Studies have shown that BATF can either promote effector function or drive T cell exhaustion, depending on the specific immune context 29, 47, 56, 146-148.

In viral infection models, chronic HIV infection has been shown to elevate BATF expression in T cells, leading to exhaustion and impairing the body's antiviral response 147. Interestingly, some other research also indicates that elevated BATF expression can enhance T cell effector functions in both chronic and acute viral infections. Xin *et al.* demonstrated that in a Clone 13 strain of lymphocytic choriomeningitis virus (LCMV) chronic infection model, IL-21 secreted by CD4^+^ helper T cells drives sustained BATF expression in CD8^+^ T cell subsets through the STAT3 signaling pathway. BATF, together with IRF4, supports Blimp-1 expression to maintain CD8^+^ T cell effector functions 148. Haining *et al.* revealed that BATF is crucial for naïve T cell activation and differentiation into effector cells. A mouse model of acute infection with the Armstrong strain of LCMV showed that BATF deficiency impairs early T cell activation, reducing T-bet, Runx3, and Blimp-1 upregulation and limiting inflammatory cytokine receptor expression, which hinders the transition of naïve T cells into effector cells 29.

Likewise, in tumor models, the effects of BATF on T cell-mediated antitumor responses remain inconsistent and context-dependent. Seo *et al.* proved that BATF cooperate with IRF4 could steer CAR-T cells away from exhaustion and toward an effector-like phenotype 56. They showed that CAR-T cells overexpressing BATF exhibited increased effector cytokines secretion, enhanced proliferation, decreased levels of exhaustion-associated TOX, increased expansion potential and tumor rejection. Additionally, BATF serves as a crucial target of Regnase-1, which dose-dependently suppresses the 3' UTR of *Batf*, thereby influencing mitochondrial fitness and effector functions 146. Regnase-1 deletion reprogrammed T cells to enhance BATF function and mitochondrial metabolism, resulting in greater T cell persistence and survival, thereby improving antitumor effectiveness in both blood and solid tumor models. However, Zhang *et al.*, using a CAR-T exhaustion model induced by chronic antigen stimulation combined with clustered regularly interspaced short palindromic repeats (CRISPR) screening, demonstrated that BATF promotes T cell exhaustion via direct binding to exhaustion-associated genes and increasing their expression 47. Knocking out BATF, rather than overexpressing it, upregulated T cell activation-associated genes while suppressing exhaustion-related genes, promoting the differentiation of central memory T cells, and enhancing tumor rejection.

A deeper investigation into the conflicting effects of BATF on T cell differentiation and function across studies is crucial for achieving a more precise and effective optimization of CAR-T therapy.

Zhang found that the differences in tumor-killing models employed in these studies play a significant role. In Zhang's study, BATF was shown to drive T cell exhaustion under conditions of low T cell-to-tumor cell ratios and repeated tumor antigen stimulation, which is an exhaustion-inducing microenvironment. In this context, BATF deficiency reduced CAR-T exhaustion and enhanced their cytotoxicity. They also found that in a non-exhaustion context characterized by a high CAR-T-to-tumor ratio and transient tumor stimulation, BATF knockout had no impact on exhaustion or memory formation, while BATF overexpression enhanced CAR-T cytotoxicity, which aligns with Seo *et al.*'s observations 47. Notably, Seo's study utilized a short-term co-culture cytotoxicity assay without repeated tumor antigen stimulation, minimizing exhaustion-inducing factors 56. However, the conclusion by Zhang *et al.* that the exhaustion context determines BATF's regulation of T cell function appears to be inaccurate. As previously noted, even in chronic viral infection models under exhaustion conditions, BATF's impact on T cell exhaustion varies significantly and is sometimes contradictory, as observed in studies of chronic HIV infection and Xin *et al.*'s chronic infection model 147, 148.

Further exploration of the regulatory mechanisms of BATF in T cell function is needed. Studies have reported that in infection models, CD8^+^ T cells differentiate into three distinct subsets: progenitors (Ly108⁺TCF-1⁺), terminally exhausted (Ly108⁻CX3CR1⁻) and cytotoxic effector cells (CX3CR1⁺) 149, 150. Chen *et al.* investigated the gene regulatory networks of these subsets and found that BATF expression induced by chronic infection binds to the regulatory regions of *Tbx21* and *Klf2*, enhancing their expression and facilitating the transition of TCF-1⁺ progenitor cells into CX3CR1⁺ effector cells 57. This study suggests that increasing BATF expression may drive the differentiation of progenitor T cells into CX3CR1⁺ effector cells, boosting CD8⁺ T cell effector functions. However, as chronic infection progresses, Chen *et al.* observed that terminally exhausted CD8⁺ T cells gradually increase 30 days after LCMV infection 57. At this stage, BATF overexpression no longer enhances effector functions but may instead collaborate with IRF4 and NFAT to drive exhaustion 140.

Therefore, the discrepancies in BATF's effects on T cell differentiation and function across studies may stem from differences in the stages of T cell differentiation (progenitor-effector-exhaustion) induced by chronic stimulation in different models. For example, in the HIV infection model, chronic T cell exhaustion persists for several months or years, while in the LCMV chronic infection model, exhaustion occurs within 30 days. The latter contains a higher proportion of progenitor T cells, allowing BATF overexpression to enhance effector functions.

Additionally, the cytokine composition during CAR-T culture plays a pivotal role in shaping T cell metabolism and T cell exhaustion phenotypes. For instance, the addition of IL-21 into culture promotes oxidative phosphorylation, reduces BATF expression, and inhibits exhaustion, whereas IL-2 and IL-12 synergistically induce a glycolytic metabolic shift, favoring exhaustion phenotypes 151. What's more, high IL-2 doses are known to upregulate STAT5, activate tryptophan hydroxylase 1, and drive aryl hydrocarbon receptor nuclear translocation, resulting in T cell exhaustion 152. The cytokines used in CAR-T culture procedures vary among these studies. Zhang's study used a high IL-2 dose (400 IU/mL) for CAR-T culture 47, in contrast to Seo's 100 IU/mL 56, which potentially increased the likelihood of T cell exhaustion, which may have further contributed to the observed differences in the role of BATF.

In conclusion, BATF's role in CAR-T cells is highly context and T cell differentiation stage dependent. Understanding these factors is crucial for harnessing BATF's potential in CAR-T therapy. Further mechanistic research is necessity to better understand how to regulate BATF for optimized applications in CAR-T therapy.

#### 3.2.2 TOX and NR4A

The TOX protein is part of the HMG-box family and interacts with DNA in a sequence-nonspecific fashion 153, 154. TOX plays a vital role in the ontogeny of innate lymphoid cells, NK cells, and T cells 155-157. In 2019, Alfei *et al.* found that TOX decreases T cell effector function and drives, as well as sustains exhaustion status during persistent infections 156. Further research has demonstrated that TOX orchestrates transcriptional networks associated with T cell exhaustion by altering chromatin accessibility and modulating the activity of gene enhancers and promoters, ultimately driving irreversible exhaustion 158.

TOX is upregulated during prolonged stimulation and is highly expressed in CD8^+^ TILs due to sustained tumor antigen interactions 48, 159, 160. This makes TOX a valuable biomarker for assessing T cell exhaustion and predicting patient responses to anti-PD-1 immunotherapy 58, 59. In hepatocellular carcinoma (HCC), TOX facilitates PD-1 endocytic recycling, thereby resulting in sustained high levels of PD1 expression. TOX downregulation led to increased T cell infiltration, reduced dysfunction and an improved response to anti-PD1 therapy 59. Targeting TOX has shown promise in enhancing T cell infiltration and reversing dysfunction in exhausted T cells.

Similarly, the NR4A family of orphan nuclear receptors, including NR4A1, NR4A2, and NR4A3, are crucial mediators of T cell exhaustion. Knockout of *NR4a1/2* increases the proportion of TCF1^+^ exhaustion precursor CD8^+^ T cells, boosts both glycolysis as well as oxidative phosphorylation, and enhances T cell persistence within the tumor microenvironment 60. This makes NR4A an appealing target for reinvigorating CAR-T cells by promoting early memory differentiation while limiting exhaustion.

TOX and NR4A are both integral components of the TCR (T cell receptor) -NFAT (nuclear factor of activated T cells)-TOX/NR4A axis, a critical signaling pathway that governs T cell fate upon TCR activation 161, 162. Under conditions of chronic TCR/CAR stimulation, limited AP-1/NFAT interactions would induce a hyporesponsive state in T cells 163. Thus, NFAT initiates a cascade that drives the expression of TOX and NR4A. This pathway forms a positive feedback loop in which TOX and NR4A enhance each other's expression, reinforcing inhibitory receptors expression and chromatin accessibility remodeling, ultimately hampering T cell function 48. While NFAT plays a central role in orchestrating this axis, its broad involvement in T cell activation and differentiation complicates its potential as a therapeutic target 163-165. For instance, theoretically reducing NFAT activity could mitigate exhaustion by attenuating TOX/NR4A expression; however, NFAT knockout has been proved to impair T cell memory differentiation and diminish effector function 166, 167.

**Figure 6:**
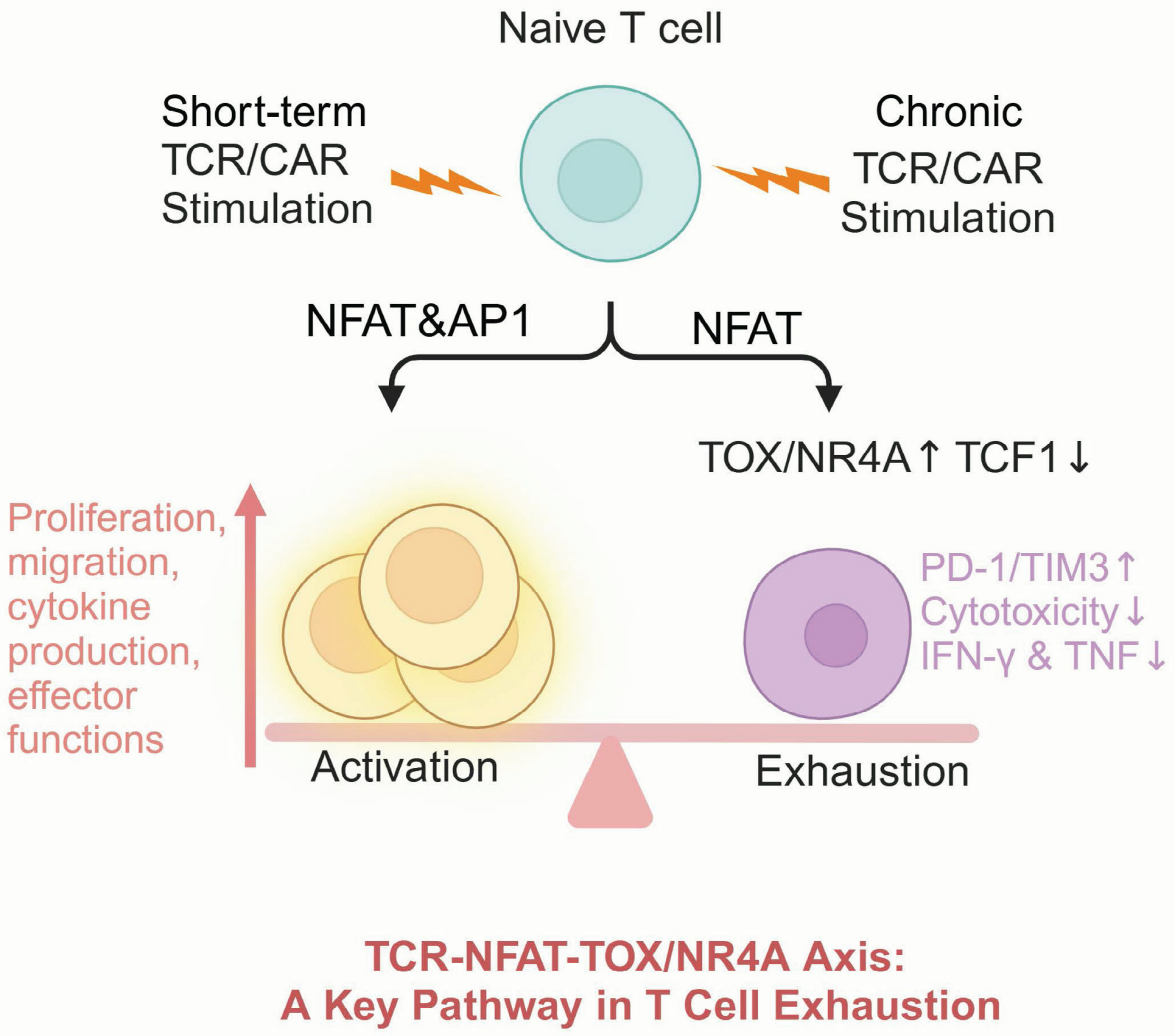
** T cell exhaustion is regulated through the TCR-NFAT-TOX/NR4A pathway.** Upon TCR activation through antigen recognition, downstream AP-1 expression is upregulated, and together with NFAT, which promotes the transcription of T cell effector genes. Under chronic TCR stimulation, the intracellular levels of NFAT and AP-1 decrease, leading to increased expression of TOX and NR4A, suppression of TCF1, and promotion of the exhausted T cell phenotype. Figure was created in BioRender.com.

Thus, directly targeting NFAT could inadvertently disrupt essential immune functions, making it an unsuitable candidate for precise modulation. Recent attention has shifted to TOX and NR4A as actionable targets within the TCR-NEAT-TOX/NR4A axis (**Figure 6**). Targeting TOX and NR4A in CAR-T therapy offers a viable method to improve T cell durability and functionality.

Seo *et al.* showed that TOX protein is highly induced in dysfunctional CAR-T cells which expression closely correlated with higher PD-1 expression and impaired cytolytic activity and cytokine production 48. CAR-T cells with dual genetic ablation of TOX and TOX2 exhibited improved antitumor activity and downregulation of inhibitory receptors. In contrast to their wild-type counterparts, TOX/TOX2-KO CAR-T cells revealed attenuated expression of *Nr4a1* and *Nr4a2*, indicating the underlying interactions among NFAT, NR4A, and TOX in driving T cell exhaustion.

Chen and colleagues also explored the function of NR4A TFs in CAR-T cells 50. Consistent with other studies, they found that NR4A family members show significant upregulation in exhausted CAR-T cells, with their expression positively correlates with PD-1 and TIM3 levels. NR4A triple KO CAR-T cells exhibited enhanced effector potential and greater tumor control capabilities.

Taken together, these findings highlight the prominent role of the TCR-NFAT-TOX/NR4A axis in mediating CAR-T dysfunction and underscore the therapeutic potential of targeting TOX and NR4A to improve CAR-T efficacy in solid tumors.

#### 3.2.3 Blimp-1

B lymphocyte-induced maturation protein-1 (Blimp-1), which is encoded by the PR domain zinc finger protein-1 (*PRDM1*), acts as a key epigenetic factor in T-cell exhaustion 168, 169. Blimp-1 has been reported to induce terminal differentiation in both B and T cell lineages and is recognized as a key suppressor of memory T cells during viral infections 49, 61. It is also associated with the formation of short-lived effector cells 31 and has been implicated in immune suppression within acute myeloid leukemia (AML) 62. In T cells from AML patients, Blimp-1 is upregulated and associated with terminally differentiated effector T cells. These Blimp-1^+^ T cells exhibit functional defects, such as reduced cytokine production and impaired cytotoxicity. Moreover, Blimp-1 directly upregulates PD-1 and TIGIT expression through specific binding with their promoters.

**Figure 7:**
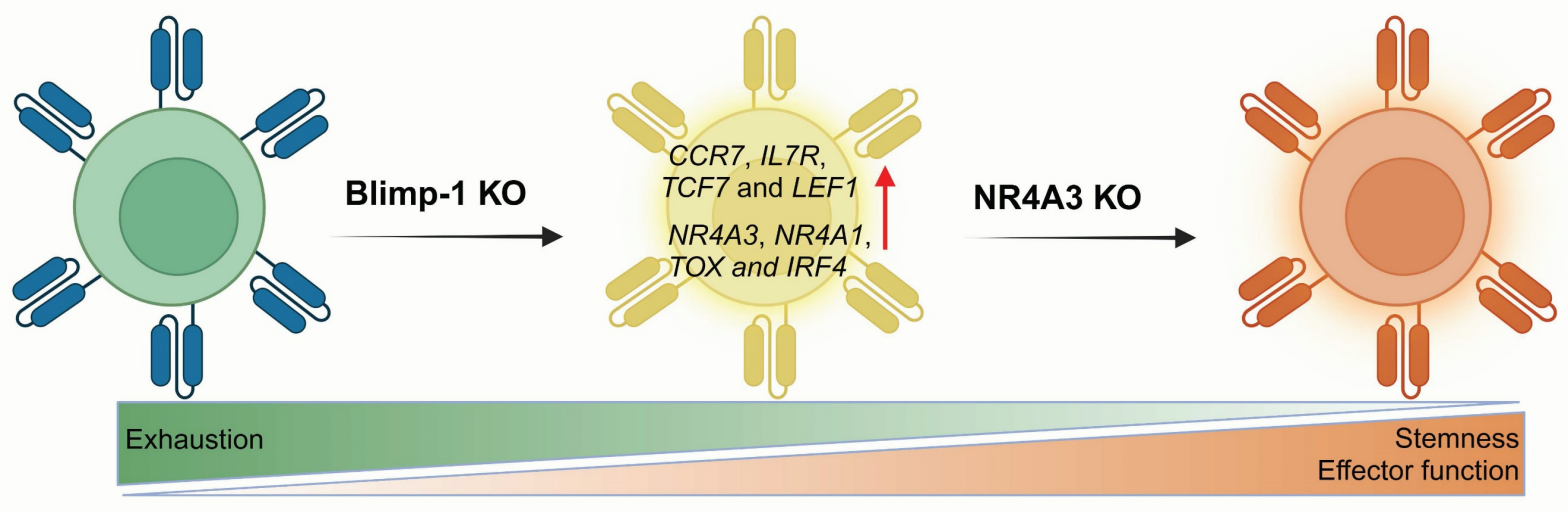
** Blimp-1 is involved in the regulation of T cell memory differentiation.** Compared to Blimp-1 deletion alone, dual knockout of Blimp-1 and NR4A3 synergistically reduces T cell exhaustion and enhances antitumor responses across multiple murine models, yielding superior outcomes. Figure was created in BioRender.com.

In 2022, Oshikawa *et al.* examined how PRDM1 knockout affects the characteristics of CD19 CAR-T cells 63. They found that PRDM1 deficiency could help maintain the memory phenotype and enhance cytokine polyfunctionality as well as the therapeutic response. Genome-wide gene expression profiling revealed that PRDM1-knockout CAR-T cells markedly increased the expression of memory-associated genes, including *CCR7*, *IL7R*, *TCF7* and *LEF1*. However, they also noted an increase in TOX expression, which is relative to T cell exhaustion. Subsequently, Jung and colleagues investigated the potential of PRDM1 knockout to boost the efficacy of CAR-T therapy in solid tumors 64. Although PRDM1 single knockout enhanced early memory differentiation and CAR-T cell polyfunctionality, which was driven primarily by TCF7 upregulation. *In vivo* experiments showed that PRDM1 knockout only slightly improved leukemia control in mice. Further research revealed that PRDM1 deficiency triggered negative feedback driven by enhanced calcineurin-NFAT signaling, leading to T cell dysfunction. This process triggers compensatory upregulation of several T cell exhaustion genes such as *NR4A3*, *NR4A1*, *TOX* and *IRF4*, which diminishs the antitumor effectiveness of T cells. They further knocked out NR4A3, the most significantly upregulated gene in the feedback loop, confirming that dual PRDM1/NR4A3 knockout boosts long-term antitumor activity by preserving both stemness and effector function, achieving superior antitumor efficacy compared with that of PRDM1 deletion alone (**Figure 7**).

Therefore, targeting a single exhaustion-related molecule alone may be insufficient to fully reverse T cell exhaustion. Multiplex gene-editing strategies offer a promising approach to counteract the compensatory upregulation of exhaustion genes observed with single-gene knockouts, thereby facilitating the formation of stem-like T cell subsets and improving therapeutic outcomes.

#### 3.2.4 IKZF3

Ikaros family zinc finger 3 (IKZF3) is a member of the IKAROS family and is crucial for lymphopoiesis and differentiation 170. Additionally, IKZF3 promotes tumor progression, and its high expression in intestinal-type gastric cancer is linked to a poor prognosis 171. IKZF3 possesses four zinc finger domains, which are critical for recognizing the common DNA motif a/gGGAA. Mutations in the second zinc finger domain of IKZF3 have been identified as potential drivers of CLL. These mutations alter IKZF3's DNA-binding properties, leading to CLL transformation via excessive B-cell receptor (BCR) signaling and increased expression of NK-κB target genes 172. In mouse models, IKZF3 knockout has been demonstrated to result in B cell lymphoma formation 173.

**Figure 8:**
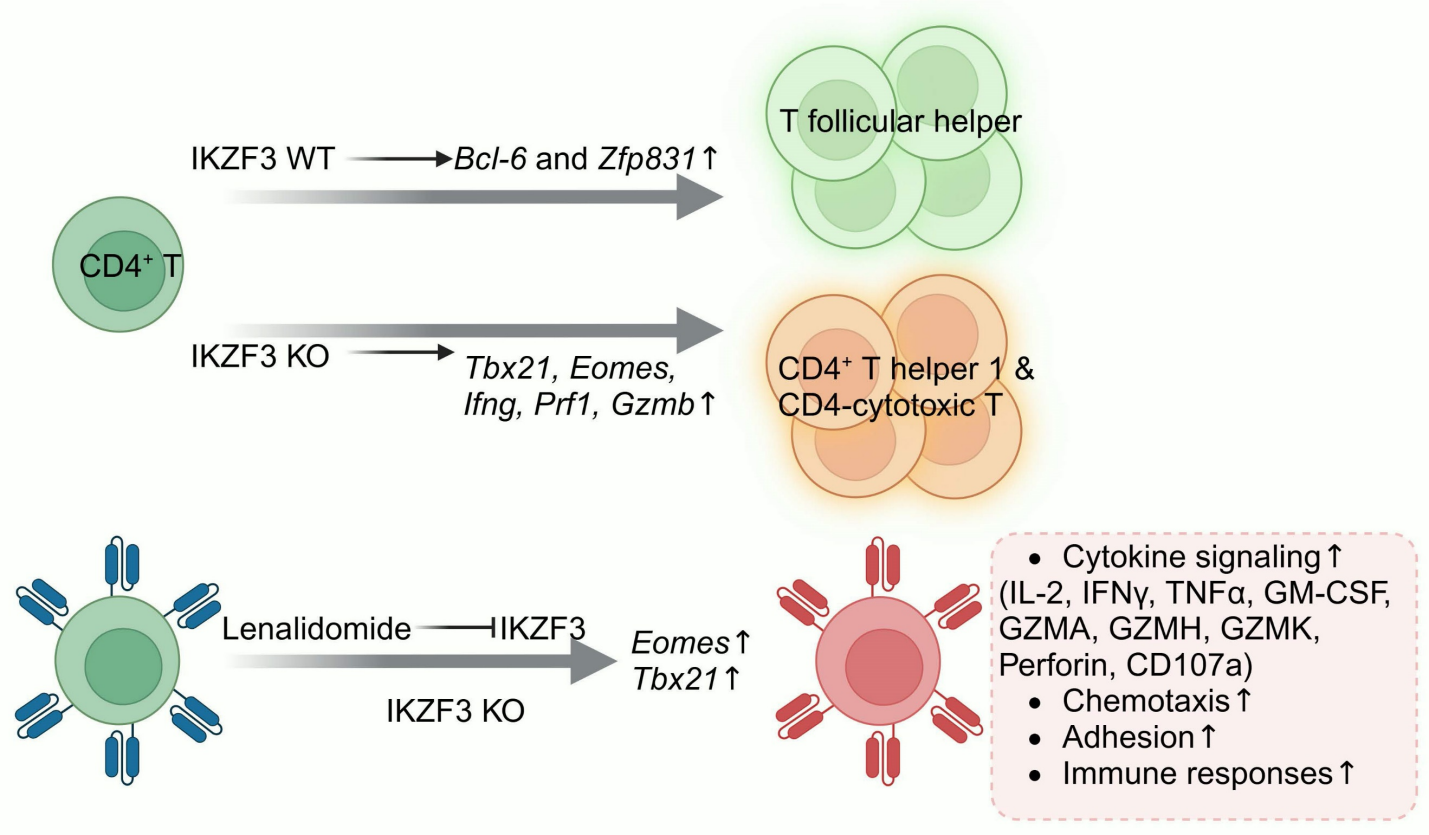
** Schematic of IKZF3 regulation in T cell differentiation.** IKZF3 promotes CD4+ T follicular helper cell differentiation. Upon IKZF3 knockout, CD4+ T cells shift toward Th1 and CD4-CTL differentiation. In CAR-T cells, treatment with lenalidomide or IKZF3 knockout enhances the transcription of Eomes and Tbx21, boosting cytokine secretion, chemotactic function, adhesion capacity, and immune responsiveness. Figure was created in BioRender.com.

Research on IKZF3 function in CD4^+^ T cells has shown that IKZF3 facilitates T follicular helper (Tfh) cell commitment by inducing the TFs Bcl-6 and Zfp831 (**Figure 8**). Knockout of IKZF3 in mice reduces the proportion of antigen-specific Tfh cells, significantly increases *Tbx21*, *Eomes*, *Ifng*, *Prf1* and *Gzmb* expression; promotes Th1 and CD4 cytotoxic T lymphocyte (CTL) differentiation; and boosts the granzyme B, IFN-γ, and perforin secretion in response to antigenic stimulation 174. Lenalidomide, an oral administered immunomodulator, has been approved by the FDA for treating multiple myeloma and myelodysplastic syndromes. Lenalidomide modulates T cell function through the induction of the ubiquitination and subsequent degradation of IKZF1 and IKZF3. Zhu *et al.* proved that lenalidomide treatment of HER2 CAR-T and CD133 CAR-T cells increased effector cytokines secretion, such as IL-2, IFNγ, tumor necrosis factor α (TNFα) and GM-CSF, and increased specific cytotoxicity 65. Hay and colleagues also discovered that lenalidomide treatment reversed T cell exhaustion in CAR-T cells and enhanced their responsiveness to immune checkpoint inhibitors 66. Zhu and Chi collaborated to knock out IKZF3 in HER2 and CD133 CAR-T cells, which indeed enhanced antitumor activity. Transcriptomic analysis showed that IKZF3 knockout enhanced cytokine signaling, chemotaxis, and adhesion pathways, boosting effector molecule secretion. However, it also upregulated several pro-apoptotic genes, such as BCL2L11 and cyclin dependent kinase inhibitor 2a (CDKN2A), thus slightly increasing CAR-T cell apoptosis 67.

Overall, modulating IKZF3 expression through different approaches could improve effector function, reverse T cell exhaustion, and propose a new approach to boost CAR-T therapy efficacy against solid tumors. However, it is crucial to consider that IKZF3 also participates in the orchestration of lymphocyte development, and its knockout may pose a potential risk of tumorigenesis.

## 4. Therapeutic targeting of epigenetic regulators in CAR-T therapy

**Figure 9:**
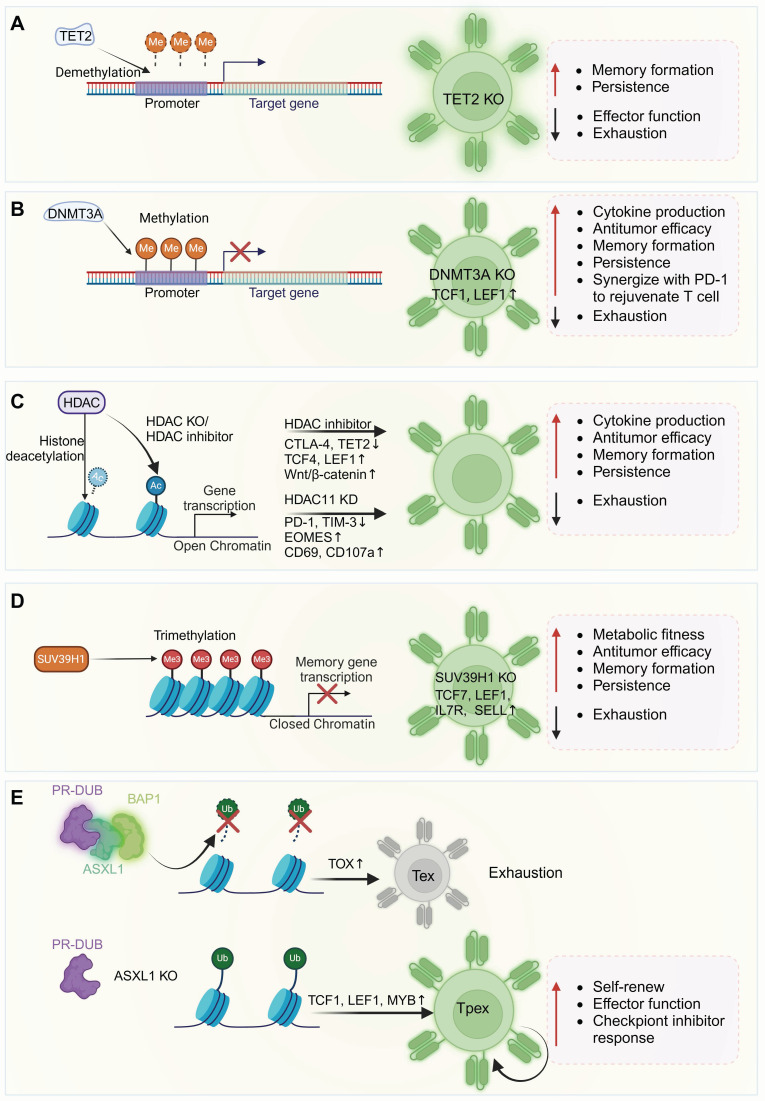
Schematic of epigenetic regulators influencing DNA and chromatin landscapes to modulate T cell differentiation and function. (A) TET2 mediates DNA demethylation, promoting target gene transcription. TET2 knockout enhances T cell memory differentiation and persistence, resisting exhaustion from chronic stimulation. (B) DNMT3A expression increases post-T cell activation, leading to Tcf7 methylation. DNMT3A knockout elevates TCF-7 and LEF1 expression, boosting T cell effector activity, memory formation, and sensitivity to anti-PD-L1 treatment. (C) HDACs facilitate the removal of acetyl moieties from lysine residues on histone and non-histone proteins, inhibiting gene transcription. HDAC inhibition or knockout enhances effector gene expression, suppresses exhaustion markers, increases the expression of the TFs Eomes, TCF4, LEF1, and activates Wnt/β-catenin signaling. (D) SUV39H1 primarily mediates H3K9me3, inducing heterochromatin formation and gene silencing. SUV39H1 knockout increases the transcription of memory-associated CAR-T genes (TCF7, LEF1, IL7R, and SELL), enhancing metabolic adaptability and effector function. (E) ASXL1 facilitates the removal of ubiquitin from H2AK119Ub. ASXL1 deficiency or knockout preserves more ubiquitinated gene sites, opening chromatin regions that support T cell stemness and functionality and enhancing T cell maintenance and responsiveness to ICB. ASXL1: additional sex combs-like 1; HDAC: histone deacetylase; PR-DUB: polycomb repressive deubiquitinase; BAP1: BRCA1 associated protein. Figure was created in BioRender.com.

As discussed above, transcription factors play pivotal roles in regulating T cell function as well as memory and exhaustion differentiation. Similarly, epigenetic modifications also exert a widespread influence over T cell biology (**Table 1**). Epigenetic modifications in T cells, such as CpG dinucleotide methylation in DNA promoter regions 175 and histone acetylation and methylation of nucleosomes, contribute to shaping T cell differentiation states and functional capacities 176, 177. Researchers have modulated T cell differentiation by focusing on epigenetic regulators, including DNA demethylases, DNA methyltransferases and histone deacetylases, etc, to support memory T cell generation while reducing or avoiding T cell exhaustion (**Figure 9**).

### 4.1 TET2

Tet methylcytosine dioxygenase 2 (TET2), a member of the TET family of epigenetic regulators, oxidizes 5-methylcytosine (5mC) in DNA, thus mediating DNA demethylation. TET2 mutations frequently occur in the hematopoietic and immune systems, leading to myelodysplasia and related myeloid malignancies 178, and approximately 15% of myeloid cancers harbor somatic TET2 mutations 179. Research on the role of TET2 in T cells revealed that after LCMV infection, TET2-deficient mice exhibited rapid expansion of memory gp33^+^ CD8^+^ T cells, indicating that TET2 critically directs CD8^+^ T cells differentiation into either effector or memory subsets 68.

Considering TET2's pivotal role in epigenetic regulation, an escalating trend in research is the exploration of strategies to modulate TET2 activity to enhance T cell-based immunotherapy. An intriguing case reported by Carl June's team involved a chronic lymphocytic leukemia patient in one CD19 CAR-T clinical trial 69. After two infusions of CTL019, the patient achieved rapid CLL cell clearance and complete remission. TCR repertoire analysis of the patient's peripheral blood CD8^+^ T cells indicated that although T cells were polyclonal early during treatment, 94% of the CD8^+^ T cells originated from a single clone by two months after the second CAR-T cell infusion.

NGS analysis uncovered a missense mutation at position 1879 (glutamic acid to glutamine) in the catalytic domain of the patient's TET2 gene, along with a loss-of-function heterozygous mutation due to CAR segment insertion in one allele. These combined mutations caused TET2 dysfunction, reshaped the epigenetic landscape of CAR-T cells, reduced or eliminated chromatin accessibility for genes related to T cell effector function and exhaustion (*IFNG, NOTCH2, CD28, ICOS,* and *PRDM1*), which promoted memory T cell development and prolonged CAR-T persistence *in vivo*. Fraietta and colleagues applied a targeted integration strategy to insert CD19 CAR into TET2, generating TET2 knockout CAR-T cells with more central memory T cells subsets. Upon chronic antigen stimulation, TET2-deficient CD8^+^ T cells showed a greater tendency to adopt a CCR7^+^ CD45RO^+^ memory phenotype with elevated TCF1 expression, preventing CAR-T cells from progressing to terminal exhaustion 70.

Notably, the Sadelain team found that TET2 knockout could improve CAR-T therapeutic efficacy, although this effect enhancement was dependent on the characteristics of the CAR. CAR-T cells containing the 4-1BB intracellular domain improved tumor treatment outcomes in mice after TET2 knockout, whereas CD28 signaling CAR-T cells exhibited no notable differences in cell differentiation or antitumor efficacy before versus after TET2 knockout. Further studies found that the enhancement of T cell memory phenotype and therapeutic efficacy due to TET2 deficiency depended on the epigenetic state of increased BATF3 expression, which ultimately led to antigen-independent sustained proliferation of TET2 biallelic knockout CAR-T cells 71. Nonetheless, it is crucial to recognize that TET2 deficiency-induced epigenetic alterations could potentially trigger somatic malignant transformation 180; therefore, careful consideration is required when TET2 knockout strategies are employed to enhance CAR-T therapy.

### 4.2 DNMT3A

DNMT3A and DNMT3B are the major *de novo* methyltransferases in mammals 181. After TCR-mediated activation triggered by antigen stimulation, DNMT3A transcription is upregulated over 30-fold, mediating T cell polarization toward Th1 or Th2 through the methylation regulation of IFN-γ and IL-4 72. Furthermore, DNMT3A-mediated *Tcf7* methylation in early effector T cells plays a critical role in regulating the proportion of memory precursor effector cells and restricting the development of TCF1^hi^ CD127^+^KLRG1^-^ memory precursor T cells 73.

Therefore, *de novo* DNA methylation programming reinforces T cell exhaustion and creates a stable internal barrier to T cell reactivation in response to PD-1 blockade. DNMT3A deficiency results in reduced methylation at the *IFNγ*, *Myc*, *Tcf7*, and *Tbx21* loci, resisting terminal differentiation and enhancing responsiveness to anti-PD-L1 therapy. Pretreatment with low doses of the DNA demethylating agent decitabine for two weeks enhanced the T cell response to PD-L1 blockade in Tramp-C2 tumor-bearing mice, inducing the expansion of tumor-infiltrating CD8^+^ T cells 74.

The research team further validated the impact of DNMT3A among several human CAR-T models targeting solid tumors 75. They demonstrated that DNMT3A deficiency enhanced CAR-T cell expansion during constant stimulation and sustained their cytokine secretion and tumor cell lysis. After chronic stimulation, DNMT3A KO CAR-T cells exhibited higher TCF1 and LEF1 expressions, with epigenetic profiles resembling those of stem-cell-like memory or naïve T cells, ultimately increasing *in vivo* antitumor activity across multiple solid tumor models.

### 4.3 HDAC

Histone deacetylases (HDACs) are a group of enzymes that catalyze removing acetyl groups from lysine residues on histone and non-histone proteins. HDACs are classified into four major classes based on sequence homology, comprising a total of 18 enzymes. HDACs are integral to a variety of physiological and pathological processes, exerting significant influence on their regulation. Recently, several studies have reported that HDAC expression or activity modification via inhibitors or genetic editing enhances CAR-T antitumor efficacy and persistence 76-78.

Treatment of B7-H3 CAR-T cells with the pan-HDAC inhibitor, vorinostat (SAHA), downregulated CTLA-4 and TET2 expression 78. Moreover, combining SAHA with B7-H3 CAR-T cells exhibited excellent tumor suppression effects in orthotopic patient-derived xenograft (PDX) and metastatic xenograft models. A high-throughput drug screening platform was employed by Zhu and colleagues to evaluate the effects of 370 epigenetic regulatory compounds on CAR-T cell dysfunction and phenotype modulation *in vitro* 76. Ultimately, they identified two inhibitors for class I HDACs, chidamide and M344. Mechanistic analysis revealed that these inhibitors decreased HDAC1 expression as well as enhanced histone H3K27 acetylation, which upregulated TCF4 and LEF1 while elevating intracellular β-catenin expression, reinforcing the Wnt/β-catenin signaling cascade. Consequently, they promoted T cell memory subset differentiation both *in vitro* and *in vivo*, counteracted T cells exhaustion, and improved tumor suppression efficacy. Zhang *et al.* knocked down HDAC11 in NKG2D CAR-T cells via shRNA interference, which significantly enhanced intracellular Eomes expression and cell expansion. Additionally, it reduced exhaustion markers (TIM-3, PD-1) expression, facilitated the formation of Tcm subsets, and prolonged the survival in prostate cancer-bearing mice 77.

It is important to note that HDACs are widely involved in various cellular physiological processes 182; therefore, strategies that enhance CAR-T therapy by targeting HDACs carry certain risks. Targeting HDAC inhibition may produce direct toxicity; for example, as reported by Lei *et al.*, the pan-HDAC inhibitor SAHA has shown dose-limiting toxicity in clinical trials 183, although they used low-dose SAHA to treat CAR-T cells, achieving a balance between therapeutic efficacy and the drug's safe dosage is challenging because lower doses may reduce efficacy, whereas higher doses increase toxicity 184, 185. Employing selective HDAC inhibitors or targeted genetic modifications to regulate the expression or function of specific HDACs strengthens targeting specificity to some extent, helping to reduce the side effects caused by pan-HDAC modification. However, long-term monitoring of cellular developmental safety remains necessary.

### 4.4 SUV39H1

The histone methyltransferase suppressor of variegation 3-9 homolog 1 (SUV39H1) is primarily responsible for the trimethylation of histone H3 at lysine 9 (H3K9me3), which induces heterochromatin formation and gene transcriptional silencing 186-189. SUV39H1 is pivotal in modulating T cell activity and lineage commitment, which suppresses memory- and stemness-related genes in terminal differentiation effector CD8^+^ T cells. Depletion of SUV39H1 in mice leads to reduced H3K9me3 levels in CD8^+^ T cells, which manifests as diminished production of effector cytokines (granzyme B and IFN-γ), impaired proliferation, and compromised elimination of *Listeria* infection. Additionally, the reduced gene methylation including Il7r (CD127), Sell (CD62L), Ccr7, and Cxcr4, leads to increased transcription, contributing to an increased fraction of stem-like memory T cells 190. While SUV39H1 knockout impairs T cell effector response during infection, Niborski further found that in tumor models of B16F10-OVA and MCA-101 fibrosarcoma, SUV39H1-deficient mice demonstrated stronger antitumor activity in tumor-infiltrating lymphocytes (TILs) when combined with PD-1 ICB, compared to wild-type controls. SUV39H1 deficiency appeared to delay or prevent TIL exhaustion 79.

Jain *et al.* reported that SUV39H1 knockout in human CAR-T cells led to a slight reduction in the production of effector cytokines such as TNFα, IFNγ, IL2 and granzyme B. However, after multiple rounds of tumor cell co-culture, SUV KO 1928z CAR-T cells exhibited enhanced mitochondrial activity and improved metabolic fitness, while maintaining chromatin accessibility at loci (*KLF2, LEF1, TCF7*) associated with T cell memory and effector functionality and decreasing accessibility at effector function and immune checkpoint gene loci. These modifications promoted CAR-T cell proliferation and sustained functionality under chronic antigen stimulation, reduced the proportion of exhausted subsets, and ultimately improved the antitumor efficacy and prolonged protection of CAR-T cells 80.

The effects of SUV39H1 knockout on T cell effector functions have demonstrated variability across studies, likely due to differences in disease models (tumor vs. virus), T cell activation methods (TCR vs. CAR), and therapeutic approaches (PD-1 blockade vs. T cell therapy). Overall, SUV39H1 deficiency leads to increased chromatin accessibility in T cells, particularly at loci related to memory differentiation genes such as *TCF7*, *LEF1*, *IL7R*, and *SELL*, thereby promoting the differentiation of memory T cell subsets and supporting sustained T cell effector function. This finding offers a novel therapeutic strategy for enhancing CAR-T antitumor effectiveness.

Notably, while Jain *et al.* observed no signs of malignant expansion or transformation in SUV39H1-knockout CAR-T cells during a 200-day monitoring period 80, Niborski found that when Suv39h1-KO T cells were transplanted into B6xDBA/2 F1 recipient mice, they experienced more severe graft-versus-host disease (GvHD) compared to those receiving WT B6 bone marrow. In fact, 78% of the mice infused with Suv39h1-deficient T cells died from GvHD 79. Therefore, further studies with longer durations are required to confirm the safety of SUV39H1 knockout with respect to the potential for excessive T cell proliferation.

### 4.5 ASXL1

The epigenetic regulator additional sex combs-like 1 (ASXL1) plays a key role in physiological processes. It stabilizes the polycomb repressive deubiquitinase (PR-DUB) complex and facilitates the recruitment of its partner, BRCA1 associated protein (BAP1), to mediate the deubiquitination of histone H2A at lysine 119 (H2AK119Ub). ASXL1 mutations are frequently detected in various myeloid malignancies 191, 192.

In a clinical trial conducted by O'Connell *et al.*, which combined PD-L1 immune checkpoint inhibitors with the hypomethylating agent guadecitabine in relapsed or refractory myelodysplastic syndrome (MDS) and chronic myelomonocytic leukemia (CMML) patients, a correlation was noted between long-term survival in MDS patients and the presence of ASXL1 mutations in their T cells 193. Building on this observation, Kang *et al.* recently investigated the impact of ASXL1 in regulating the gradual transfer of multipotent progenitor exhausted T cells to terminally differentiated T cells 81. The study revealed that ASXL1-knockout T cells generated a larger population of TCF1^+^ stem-like cells in a chronic LCMV infection model, resulting in more than 1 year of long-term persistence, enhanced effector function, and lower levels of the exhaustion TF Tox. However, ASXL1 does not rely on DNA methylation to regulate CD8^+^ T cell differentiation, as ASXL1-deficient T cells displayed no significant site-specific or genome-wide methylation differences compared to wild-type T cells. However, ATAC-seq revealed >5200 differentially accessible regions between ASXL1 KO and WT CD8^+^ T cells, particularly with increased accessibility to stem and memory-related genes like *Tcf7*, *Lef1* and *Myb* in ASXL1-deficient T cells. Additionally, CD8^+^ T cells with Asxl1 knockout presented elevated levels of H3K9ac, H3K4me3 and H3K27me3. The histone profile of ASXL1 KO CD8^+^ T cells closely matched that of Tpex and Teff subsets. Furthermore, ASXL1 loss destabilized the PR-DUB complex, reducing its deubiquitination function and leading to higher H2AK119Ub levels in ASXL1 KO P14 T cells compared to WT P14 T cells. In mouse tumor models, Asxl1-deficient T cells exhibited strong effector activity and responsiveness to anti-PD-L1 treatment, contributing to improved tumor control. Among cancer patients receiving ICB therapy, those with ASXL1 insertion mutations showed longer survival, as ASXL1 disruption provided CD8^+^ T cells with a selective survival benefit after PD-L1 treatment.

Thus, targeting ASXL1 offers considerable potential for enhancing the prolonged survival and enduring efficacy of T cell therapies.

## 5. Perspective

Optimizing CAR-T therapeutic effects through transcriptional and epigenetic modulation has shown substantial translational potential. Gene editing and small molecule inhibitors allow precise modulation of key regulators, reshaping gene networks involved in T cell memory differentiation and effector functions in preclinical models. The optimization strategies primarily focus on two directions. The first involves enhancing and sustaining T cell effector functions to improve tumor cell killing, as seen in c-Jun overexpression, which enhances T cell activation, promotes IL-2 and IFN-γ secretion, reduces exhaustion markers, and collectively improves CAR-T therapy 43, 51. A similar strategy involves KLF2, which drives CAR-T cells toward effector subset differentiation instead of exhaustion 67. The second direction focuses on promoting T cell memory differentiation and extending persistence, both of which are critical for achieving durable CAR-T responses 18-21. A notable example is TET2 deficiency, identified through clinical trials, which enhances long-term T cell persistence. CAR-T cells lacking TET2 tend to differentiate into CCR7⁺ CD45RO⁺ memory subsets 69. Additionally, FOXO1 overexpression can also enhance CAR-T stemness, prolonging T cell persistence 45.

It is crucial to recognize that these optimization strategies are double-edged swords. Besides the improved CAR-T efficacy, those strategies may also introduce side effects and risks that must be addressed to enable successful clinical trials. First, the enhanced functionality of modified CAR-T cells may elevate the risk of "on-target, off-tumor" toxicity. For example, in clinical trials of c-Jun-overexpressing CAR-T cells 51, 96, while c-Jun boosts CAR-T cytotoxicity, it also increases cytokine secretion and activation signals, potentially leading to CRS and unintended damage to normal tissues expressing low levels of tumor-associated antigens. Moreover, owing to inherent differences between preclinical mouse models and humans, many preclinical CAR-T data derived from mice have limited applicability in humans. Differences in antigen expression and the higher tolerance of mice to toxicities compared to humans make it difficult to predict CRS and "on-target, off-tumor" side effects. Mitigating these risks requires a comprehensive evaluation of human antigen expression patterns and distribution across tissues using tools like single-cell RNA sequencing, combined with the cautious selection of CAR-T targets to minimize or avoid potential toxicities 194.

Another widely discussed challenge is the tumorigenic risk of CAR-T cells, as gene editing could potentially introduce somatic mutations leading to malignant transformation. Theoretically, the gene editing steps in CAR-T therapy preparation may introduce random mutations. As a result, the FDA has issued a boxed warning for approved BCMA- and CD19-targeting CAR-T therapies due to the risk of T cell malignancy post CAR-T therapy 195. Interestingly, follow-up studies revealed that the incidence of second primary malignancies (SPMs) among CAR-T-treated cancer survivors was statistically comparable to that observed with standard therapies 196. Additionally, a recent analysis of a French CAR-T patient database reported that among 3,066 patients treated with CAR-T and followed for up to 17.7 months, only one case of T cell malignancy occurred, representing an incidence rate of 0.03%. This indicates that the risk of malignant transformation from random mutations is very low 197. However, transcription factors like IKZF3 and TET2, due to their roles in T cell lineage commitment and tumorigenesis, may theoretically pose a higher risk of tumor development when manipulated 173, 178, 179. While no definitive evidence of tumorigenesis has been observed in current studies on TET2- and IKZF3-modified CAR-T cells, rigorous post-treatment monitoring of CAR-T pharmacokinetics and gene editing outcomes remains critical to identify and manage potential malignant transformations. In summary, comprehensive risk assessments and proactive safety measures tailored to the mechanisms and characteristics of CAR-T modifications are essential to ensure the clinical safety of these enhanced therapies.

Furthermore, as part of translational research, the feasibility of producing GMP-grade CAR-T therapies must be evaluated after proof-of-concept validation and before transitioning to human studies. Fortunately, existing GMP-grade CAR-T production technologies can accommodate both gene overexpression and knockout modifications 198. Thus, production-related challenges are currently within a solvable scope.

However, it is important to recognize that the transcriptional and epigenetic targets discussed in this review exhibit diverse mechanisms of action and play broad roles in regulating T cell development, function, and differentiation. The intricate interactions between these targets and their downstream signaling pathways are not yet fully elucidated. Additionally, the limited availability of real-world clinical trial data makes it challenging to identify the most effective optimization strategy for enhancing CAR-T efficacy. Further mechanistic studies are still needed to establish a comprehensive, precise, and safe foundation for future clinical trials.

## Figures and Tables

**Table 1:** Summary of transcriptional and epigenetic regulators involved in T cell effector functions and memory formation

Transcriptional and Epigenetic Modifications	Regulators	Biological roles	Interventions	Impacts on T cells	Refs.
**Memory relative TFs**	c-Jun	Correlates with various biological processes Drives T cell activation genes expression Enhances T cell cytotoxicity and effector functions	Overexpression	Enhances cytokine secretion Promotes memory phenotypes Diminishes exhaustion	43, 51
	TCF7	Involves in Wnt signaling regulation Correlates with T cell development and differentiation	Overexpression	Promotes memory differentiation Diminishes exhaustion	40, 52
	FOXO1	Participants in regulating cellular processes Regulated by PI3K-AKT signaling pathway Regulates the homeostasis and survival of naïve T cells and functions of dendritic cell, macrophages and B cells	Overexpression	Promotes memory differentiation Diminishes exhaustion Boost CAR-T cell expansion and persistence Enhances cytokine secretion	44, 45
	STAT5	Activity is predominantly regulated by IL-2 cytokine family Regulates T cell homeostasis and immune function Rejuvenates exhausted CD8^+^ T cells by counteracting TOX	Overexpression	Diminishes exhaustion Boost CAR-T cell expansion and persistence Enhances cytokine secretion	53-55
	FOXP1	Correlates with immune responses, organ development and cancer pathogenesis Regulates the quiescence and homeostasis of naïve T cells	Knockout	Impairs tumor suppression Diminishes proliferative capacity Downregulates stemness-associated genes Regulates checkpoints of stem-like to effector transition in CAR T cells	46
	KLF2	Participants in regulating cellular processes Correlates with functionality and homeostasis of immune cells and T cells migration	Knockout	Impairs effector differentiation, Enhanced the exhaustion program and upregulated Tox Regulates checkpoints of stem-like to effector transition in CAR T cells	46
**Exhaustion-inducing TFs**	BATF	Negatively regulates AP-1-mediated T cell effector functions Drive the differentiation of progenitor T cells into CX3CR1⁺ effector cells The effects on T cell-mediated antitumor responses remain inconsistent and context dependent.	Overexpression or Knockout	Represents dual Effect on CAR-T cells Correlates with T cells exhaustion, memory differentiation, and cytotoxicity.	47, 56, 57
	TOX	Correlates with ontogeny of innate lymphoid cells, NK cells, and T cells Regulates T cells dysfunction	Knockout	Increases cytokine expression Decreases expression of inhibitory receptors Enhances the cytolytic activity	58, 59
	NR4A	Participants in regulating cellular processes Regulates T cells dysfunction Associates with chronic inflammation, altered immune cell responses, and cancer development.	Knockout	Decreases inhibitory receptor expression Increases cytokine production Enhances effector functions	50, 60
	Blimp-1	Induces terminal differentiation in both B and T cell lineage Associates with immune suppression and formation of short-lived effector cells	Knockout	Maintains the memory phenotype Enhances cytokine polyfunctionality	49, 61-64
	IKZF3	Participants in lymphopoiesis and differentiation Promotes tumor progression Facilitates T follicular helper (Tfh) cell commitment	Knockout	Enhances cytokine signaling, chemotaxis, and adhesion pathways Boosts effector molecule secretion Reverse T cell exhaustion	65-67
**Epigenetic regulator**	TET2	Mediating DNA demethylation Related to myeloid malignancies Directs T cells differentiation	Knockout	Promotes memory differentiation Diminishes exhaustion Increases CAR-T persistence	68-71
	DNMT3A	*De novo* methyltransferases Mediates T cell Th1 and Th2 polarization Regulates the proportion of memory precursor effector cells	Knockout	Promotes memory differentiation Enhances CAR-T cells expansion Increases cytokine production	72-75
	HDACs	Removing acetyl groups from lysine residues on histone and non-histone proteins Participants in a variety of physiological and pathological processes Involves in various cellular physiological processes	HDAC inhibitor	Promotes memory differentiation Diminishes exhaustion Enhances CAR-T cells expansion	76-78
	SUV39H1	Histone lysine methyltransferase, induces heterochromatin formation and gene transcriptional silencing Modulates T cell activity and lineage commitment Promotes the differentiation and expansion of effector T cells	Knockout	Promotes memory differentiation Diminishes exhaustion Optimizes the long-term functional persistence	79, 80
	ASXL1	Participants in physiological processes Related to myeloid malignancies Regulates T cell differentiation	Knockout	Promotes memory differentiation Diminishes exhaustion Synergized with anti-PD-L1 blockade	81

## Abbreviations

AICE: AP-1-IRF composite elements
AML: acute myeloid leukemia
AP-1: activator protein 1
ASXL1: additional sex combs like 1
ATAC-seq: assay for transposase-accessible chromatin using sequencing
ATF: activating transcription factor
BAP1: BRCA1 associated protein 1
BATF: basic leucine zipper atf-like transcription factor
BCL2L1: BCL2-like 1
Bcl6: B-cell lymphoma 6
BCMA: B-cell maturation antigen
BCR: B-cell receptor
Blimp-1: B-lymphocyte-induced maturation protein 1
bZIP: basic leucine zipper domain protein
CAR-T: chimeric antigen receptor t-cell therapy
CCR7: C-C chemokine receptor type7
CDKN2A: cyclin dependent kinase inhibitor 2a
CLL: chronic lymphocytic leukemia
CMML: chronic myelomonocytic leukemia
CRISPR: clustered regularly interspaced short palindromic repeats
CRS: cytokine release syndrome
CTL: cytotoxic t lymphocyte
CTLA-4: cytotoxic t-lymphocyte-associated protein 4
CX3CR1: CX3C motif chemokine receptor 1
DFO: Deferoxamine
DNMT3: DNA (cytosine-5)-methyltransferase 3
E2A: T-cell E2A transcription factor
Eomes: Eomesodermin
ERK: extracellular signal-regulated kinase
Ezh2: enhancer of zeste homolog 2
FDA: Food and drug administration
FOS: fos proto-oncogene
FOXO1: forkhead box o1
FOXP1: forkhead box p1
G9A: histone-lysine n-methyltransferase g9a
Gata1: Gata binding protein 1
GM-CSF: granulocyte-macrophage colony-stimulating factor
GZMA: granzyme a
GZMB: granzyme b
H2AK119Ub: monoubiquitinated histone h2a at lysine 119
H3K9me3: histone h3 lysine 9 trimethylation
HAVCR2: hepatitis a virus cellular receptor 2
HCC: hepatocellular carcinoma
HDAC: histone deacetylase
HER2: human epidermal growth factor receptor 2
HMG: high mobility group
ICB: immune checkpoint blockade
ICOS: inducible t-cell costimulator
ID2: inhibitor of DNA binding 2
IFNγ: interferon γ
IKZF3: Ikaros family zinc finger 3
IL2RA: interleukin 2 receptor alpha
IL-7R: interleukin 7 receptor
IRF4: interferon regulatory factor 4
ISG: interferon-stimulated gene
JUN: Jun proto-oncogene
KLF2: Kruppel-like factor 2
KLRC1: killer cell lectin like receptor c1
KLRD1: killer cell lectin like receptor d1
KLRE1: killer cell lectin like receptor e1
KLRG1: killer cell lectin like receptor g1
LAG3: lymphocyte activation gene 3
LCMV: lymphocytic choriomeningitis virus
LEF1: lymphoid enhancer-binding factor 1
MAF: musculoaponeurotic fibrosarcoma oncogene
MDS: myelodysplastic syndromes
MEK: mitogen-activated protein kinase kinase
MM: multiple myeloma
NFAT: nuclear factor of activated t cells
NF-KB: nuclear factor kappa-light-chain-enhancer of activated b cells
NGS: next-generation sequencing
NR4A: nuclear receptor subfamily 4 group a
OXPHOS: oxidative phosphorylation
PD-1: programmed cell death protein 1
PI3K: phosphoinositide 3-kinase
PRDM1: PR domain zinc finger protein 1
PR-DUB: polycomb repressive-deubiquitinase complex
PRF1: perforin 1
ROR1: receptor tyrosine kinase-like orphan receptor 1
RUNX3: Runt-related transcription factor 3
S1P: sphingosine-1-phosphate
scRNA-seq: single-cell RNA sequencing
SELL: L-selectin
SPMs: second primary malignancies
SREBP2: sterol regulatory element binding protein 2
STAT: signal transducer and activator of transcription
SUV39H1: suppressor of variegation 3-9 homolog 1
T-BET: T-box expressed in T cells
TCF1: T cell factor 1
TCR: T cell receptor
Teff: Effector T cell
TET2: Tet methylcytosine dioxygenase 2
TF: transcription factor
Tfh: Follicular helper T cell
Th: T helper cell
TIL: tumor-infiltrating lymphocyte
TIM-3: T cell immunoglobulin and mucin-domain-containing-3
TNF: tumor necrosis factor
TOX: thymocyte selection-associated high mobility group box
Tpex: progenitor-exhausted t cell
ZEB2: zinc finger e-box binding homeobox 2
Zfp831: zinc finger protein 831
